# Predicting the presence of tinnitus using ecological momentary assessments

**DOI:** 10.1038/s41598-023-36172-7

**Published:** 2023-06-02

**Authors:** Marius Breitmayer, Michael Stach, Robin Kraft, Johannes Allgaier, Manfred Reichert, Winfried Schlee, Thomas Probst, Berthold Langguth, Rüdiger Pryss

**Affiliations:** 1 Institute of Databases and Information Systems, Ulm University, Ulm, Germany; 2 Institute of Clinical Epidemiology and Biometry, University of Würzburg, Würzburg, Germany; 3 Department of Clinical Psychology and Psychotherapy, Ulm University, Ulm, Germany; 4 Institute for Information and Process Management, Eastern Switzerland University of Applied Sciences, St. Gallen, Switzerland; 5 Clinic and Policlinic for Psychiatry and Psychotherapy, University of Regensburg, Regensburg, Germany; 6 Department for Psychotherapy and Biopsychosocial Health, Danube University Krems, Krems, Austria

**Keywords:** Signs and symptoms, Computer science

## Abstract

Mobile applications have gained popularity in healthcare in recent years. These applications are an increasingly important pillar of public health care, as they open up new possibilities for data collection and can lead to new insights into various diseases and disorders thanks to modern data analysis approaches. In this context, Ecological Momentary Assessment (EMA) is a commonly used research method that aims to assess phenomena with a focus on ecological validity and to help both the user and the researcher observe these phenomena over time. One phenomenon that benefits from this capability is the chronic condition tinnitus. TrackYourTinnitus (TYT) is an EMA-based mobile crowdsensing platform designed to provide more insight into tinnitus by repeatedly assessing various dimensions of tinnitus, including perception (i.e., perceived presence). Because the presence of tinnitus is the dimension that is of great importance to chronic tinnitus patients and changes over time in many tinnitus patients, we seek to predict the presence of tinnitus based on the not directly related dimensions of mood, stress level, arousal, and concentration level that are captured in TYT. In this work, we analyzed a dataset of 45,935 responses to a harmonized EMA questionnaire using different machine learning techniques. In addition, we considered five different subgroups after consultation with clinicians to further validate our results. Finally, we were able to predict the presence of tinnitus with an accuracy of up to 78% and an AUC of up to 85.7%.

## Introduction

The ubiquity of smart mobile devices (e.g., smartphones) opens new opportunities for data collection in healthcare. Ecological Momentary Assessment (EMA)^1^ is a research method that aims to assess phenomena with a focus on ecological validity by allowing subjects and patients to report repeatedly in real time, in real environments, over time, and in different contexts, thus avoiding retrospective reporting bias^2^. The mHealth platform *TrackYourTinnitus (TYT)* uses EMA in combination with mobile crowdsensing (MCS) to track a user’s individual tinnitus and monitor and assess its variability over time^3,4^. More specifically, baseline questionnaires are first used to collect users’ sociodemographic data. Then, a dynamic EMA questionnaire (EMA-D) is used to repeatedly assess the user’s tinnitus at randomly selected times of day using eight dimensions. These dimensions include the perception (i.e., whether tinnitus is perceived at that moment) of the tinnitus (first question of the EMA-D questionnaire) in addition to its loudness and distress, as well as mood, arousal, stress, concentration, and the presence of the user’s previously reported worst symptom^4,5^. The response to the first question (i.e., perceived presence) of the EMA-D questionnaire is highly clinically relevant. Therefore, it would be strongly desirable to be able to predict the presence of tinnitus based on the other information provided by the app users. Consequently, the first question of the dynamic questionnaire is investigated in this paper and whether it can be predicted using machine learning methods.

On the further background of TrackYourTinnitus (TYT), it was launched in 2014. It follows the study design of an open observational study in which users can freely enroll. TYT is not and has not been advertised and we follow the principle of word of mouth as well as discovering the apps in the app stores. The general procedure consists of two parts after downloading the app, 3 baseline questionnaires have to be filled in at the beginning, concerning demographics and tinnitus history. Only after completing these questionnaires, users can enter the dynamic EMA questionnaires (EMA-D). Here, the user can choose to have up to 14 questionnaires randomly delivered per day or they can set the times themselves (in both cases, a notification will be sent). In addition, the questionnaire can always be self-initiated. The aim is to record the current tinnitus situation as best as possible with as many unpredictable points in time as possible. The principle has now been working very successfully since 2014 and many more of our apps have followed this principle.

Furthermore, in several studies, EMA has proven to be a highly versatile method for data collection. By collecting longitudinal data with high ecological validity, EMA is often used to gain insight into psychological and behavioral phenomena^6–8^. With regard to data assessment, a pilot study was conducted showing that EMA is an applicable method and an accepted assessment method for tinnitus data^9^. The latter is supported by the results of another study showing that emotional states influence the process of how tinnitus loudness leads to tinnitus distress^10^.

When using smart mobile devices, EMA is often combined with MCS to collect contextual data^3–5,11^. In a exploratory study, opportunistic MCS was used to collect smartphone usage logs as a proxy measure for mood monitoring^12^. The results show that highly accurate predictions based on EMA in combination with MCS are possible.

Because of the quantity of data points, longitudinal data are ideally suited for modern data analysis techniques that require large data sets. For example, longitudinal EMA data were used in one study to detect warning signs of suicidal ideation using functional linear models^13^. By collecting response times of EMA questions, another paper introduced a method for predicting short-term mood changes^14^. Similarly, EMA metadata sourced from the TYT platform was used to make predictions in a study that was able to predict users’ operating system with up to 80% accuracy^15^. While there are some machine learning approaches in the context of tinnitus, to our knowledge, there is no clear a priori model for predicting tinnitus using EMA data. Therefore, we take a data-centric approach by testing different classification models (which take different approaches to classification) to determine the model that best fits the context. In addition, the aspect of small gains in predictive power is also tracked. In the context of tinnitus, little is known about the important factors and due to the heterogeneity of the disease pattern, even small differences may play a role.

As mentioned above, the responses of the EMA-D questionnaire will be examined. In particular, the question will be answered to what extent the questions of the EMA-D questionnaire that do not assess the tinnitus experience per se (i.e., loudness and distress of the tinnitus) can be used to predict the answer to the first question, whether someone is aware of the tinnitus at a particular point in time. In addition, for the first time, the study will take into account another circumstance that has been ignored in most of the previous TYT analyses (e.g.,^16,17^: one problem with open-label observational studies using MCS and EMA such as TYT is that the frequency of responses from participating users varies widely^18,19^). There are many reasons for this. Some reasons could be elicited in interviews, such as a user feeling out of treatment and grasping at the proverbial last straw. Other reasons can only be conjectured. However, the way an app is designed and what feedback is given back to the user may play a role. Nevertheless, the paradigms of EMA and MCS in the application of medical questions are still very young, which is also reflected in the fact that there are still few results supporting the evidence. In the context of evidence, for example, it is noticeable that the WHO mERA checklist^20^ is very rarely cited or discussed. Given the variation in response behavior (i.e., frequency of responses among participating users), the prediction of the first question should consider different user groups. This is to address the question of whether the planned prediction should specifically consider different user groups and whether there are indicators to take this clinically more into account in the future.

In order to distinguish user groups, the TYT data was closely examined and threshold values were identified to divide the user groups. First, only users who completed more than 10 EMA-D questionnaires were included (*threshold 1*). Then, the upper limit of 400 completed EMA-D questionnaires was identified (*threshold 2*), which can distinguish between normal users (11-400 completed EMA-D questionnaires; referred to as *Normal Users*) and power users (more than 400 completed EMA-D questionnaires; referred to as *Power Users*). The number of EMA-D questionnaires per user are depicted in Fig. 2. Another *threshold 3* was identified whether users actually report tinnitus and non-tinnitus in the dynamic questionnaire. Here, we arrived at a further distinction of whether both values were recorded at least once for each user (labeled *Non-permanent Tinnitus Users*), or presence at least once and absence at least three times (labeled *Rather absent Tinnitus Users*). This resulted in a stratification of 5 user groups. To our knowledge, such a subdivision has not been done in other mHealth work related to tinnitus, but neither has it been done in other mHealth work in general.

Regarding the prediction of whether someone perceives tinnitus or not, another disparity in the data set is how often someone perceives one or the other status. This resulted in us having very unequal data sets not only in terms of frequency of completed questionnaires, but also in terms of classes of a user (class 0: do not perceive tinnitus, class 1: perceive tinnitus). To account for this, two approaches were taken to balance the data set. On the one hand, when possible, we automatically adjusted the weights assigned to the classes for the machine learning procedures inversely proportional to the class frequencies in the input data (we call this *balanced*) to account for the imbalance in the dataset. On the other hand, entries in the majority class were eliminated (we call this *downsampling*) to have the same number as in the minority class. These variants are also presented in the context of the results. Note that we did not add entries in the minority class (i.e., upsampling using approaches such as SMOTE^21^), as this artificially generates responses to the EMA questionnaire that would not otherwise exist.

Based on the above findings and work, in this paper, we investigate the following research questions using machine learning methods. It should be noted that for each of the predictions, we only considered independent variables (i.e., variables that are non-trivially indicative of the presence of tinnitus) to predict the presence of tinnitus for a given EMA questionnaire:

**RQ1:** To what extent can we predict the presence of tinnitus for a given EMA-D answer sheet?
**RQ2:** To what extent does the number of completed EMA-D questionnaires per user influence the prediction?
**RQ3:** To what extent does the type of tinnitus (non-permanent and rather absent) influence the prediction?

## Results

The results of the predictions are presented below. As mentioned earlier, the predictions were performed based on five user groups, so the results are presented along these user groups. As mentioned earlier, two strategies were followed to balance the dataset, and this distinction is also reflected in the results.

The eight machine learning approaches used provided different results, both in terms of the different research questions and the evaluation metrics. In summary, the results suggest that there is some relationship between the analyzed data and the presence or absence of tinnitus, which may help to better understand the causes of tinnitus.

### Results balanced

The results for the case where all users are included in the classification with the balanced approach are shown in Table 1. It is noticeable that the different classifiers are not very far apart, even though the MLP classifier performs best. About 70%-predictive power can be achieved for all users in the balanced case. The largest area under the curve is achieved by LRC.

**Table 1:** Results for all users, including standard deviation.

	All Users					
	Accuracy	F1-score	AUC	Precision	Sensitivity	Specificity
DT	0.629 (+/− 0.035)	0.645 (+/− 0.027)	0.525 (+/− 0.023)	0.788 (+/− 0.012)	0.713 (+/− 0.050)	0.336 (+/− 0.046)
RFC	0.678 (+/− 0.036)	0.674 (+/− 0.026)	0.560 (+/− 0.049)	0.788 (+/− 0.011)	0.799 (+/− 0.052)	0.257 (+/− 0.050)
SVM	0.585 (+/− 0.066)	0.615 (+/− 0.063)	0.625 (+/− 0.040)	0.833 (+/− 0.023)	0.582 (+/− 0.104)	0.596 (+/− 0.102)
CNB	0.527 (+/− 0.103)	0.555 (+/− 0.108)	0.623 (+/− 0.068)	0.826 (+/− 0.050)	0.488 (+/− 0.146)	**0.661 (+/− 0.108)**
KNC	0.733 (+/− 0.034)	**0.683 (+/− 0.022)**	0.588 (+/− 0.053)	0.777 (+/− 0.009)	0.919 (+/− 0.045)	0.091 (+/− 0.027)
LRC	0.575 (+/− 0.092)	0.604 (+/− 0.089)	**0.634 (+/− 0.047)**	**0.834 (+/− 0.036)**	0.561 (+/− 0.128)	0.624 (+/− 0.089)
MLP	**0.754 (+/− 0.024)**	**0.683 (+/− 0.012)**	0.618 (+/− 0.055)	0.777 (+/− 0.005)	**0.959 (+/− 0.035)**	0.048 (+/− 0.025)
XGB	0.678 (+/− 0.047)	0.678 (+/− 0.034)	0.597 (+/− 0.048)	0.795 (+/− 0.013)	0.787 (+/− 0.071)	0.300 (+/− 0.062)

Decision Tree (DT), Random Forest (RFC), Support Vector Machine (SVM), Complement Naive Bayes (CNB), k-nearest neighbors (KNC), Logistic Regression (LRC), Multi-layer Perceptron (MLP), Extreme Gradient Boosting (XGB)

Highest values are in bold.

The results for the *Power Users* in the case of balanced are shown in Table 2. It is noticeable that the results vary more between the classifiers in the balanced case than for all users, but that a higher prediction can also be achieved (although even higher results were expected). The largest area under the curve is provided by the SVM.

**Table 2:** Results for *Power Users*, including standard deviation.

	Power Users					
	Accuracy	F1-score	AUC	Precision	Sensitivity	Specificity
DT	0.679 (+/− 0.070)	0.701 (+/− 0.056)	0.556 (+/− 0.059)	0.841 (+/− 0.023)	0.748 (+/v 0.082)	0.367 (+/− 0.089)
RFC	0.708 (+/− 0.072)	0.718 (+/− 0.058)	0.619 (+/− 0.140)	0.837 (+/− 0.027)	0.797 (+/− 0.083)	0.305 (+/− 0.114)
SVM	0.618 (+/− 0.107)	0.657 (+/− 0.094)	**0.632 (+/− 0.183)**	**0.882 (+/− 0.070)**	0.618 (+/− 0.122)	0.614 (+/− 0.251)
CNB	0.503 (+/− 0.176)	0.540 (+/− 0.174)	0.582 (+/− 0.208)	0.825 (+/− 0.139)	0.481 (+/− 0.212)	0.603 (+/− 0.227)
KNC	0.723 (+/− 0.070)	0.725 (+/− 0.062)	0.616 (+/− 0.147)	0.835 (+/− 0.031)	0.824 (+/− 0.075)	0.267 (+/− 0.148)
LRC	0.461 (+/− 0.147)	0.498 (+/− 0.156)	0.512 (+/− 0.202)	0.818 (+/− 0.124)	0.423 (+/− 0.185)	**0.636 (+/− 0.213)**
MLP	**0.780 (+/− 0.039)**	**0.750 (+/− 0.026)**	0.580 (+/− 0.185)	0.835 (+/− 0.015)	**0.913 (+/− 0.067)**	0.178 (+/− 0.129)
XGB	0.680 (+/− 0.100)	0.703 (+/− 0.085)	0.619 (+/− 0.155)	0.845 (+/− 0.041)	0.743 (+/− 0.108)	0.393 (+/− 0.161)

Decision Tree (DT), Random Forest (RFC), Support Vector Machine (SVM), Complement Naive Bayes (CNB), k-nearest neighbors (KNC), Logistic Regression (LRC), Multi-layer Perceptron (MLP), Extreme Gradient Boosting (XGB)

Highest values are in bold.

In the cases of *Normal Users* plus balanced approach (see Table 3), the bandwidth between the results of the classifiers is again smaller, and it is also noticeable that the best prediction here is worse than in the previous two cases of *Power Users* and all users. Here, it is interesting that the XGB produces the best result the first time. LRC has the largest area under the curve.

**Table 3:** Results for *Normal Users*, including standard deviation.

	Normal Users					
	Accuracy	F1-score	AUC	Precision	Sensitivity	Specificity
DT	0.629 (+/− 0.015)	0.638 (+/− 0.013)	0.531 (+/− 0.020)	0.770 (+/− 0.009)	0.725 (+/− 0.024)	0.331 (+/− 0.043)
RFC	0.675 (+/− 0.018)	0.664 (+/− 0.016)	0.565 (+/− 0.040)	0.771 (+/− 0.010)	0.812 (+/− 0.026)	0.252 (+/− 0.046)
SVM	0.623 (+/− 0.048)	0.645 (+/− 0.042)	0.646 (+/− 0.062)	0.824 (+/− 0.038)	0.639 (+/− 0.074)	0.639 (+/− 0.074)
CNB	0.542 (+/− 0.065)	0.569 (+/− 0.066)	0.630 (+/− 0.058)	0.819 (+/− 0.030)	0.506 (+/− 0.100)	**0.655 (+/− 0.101)**
KNC	0.733 (+/− 0.029)	0.661 (+/− 0.018)	0.612 (+/− 0.047)	0.758 (+/− 0.008)	0.951 (+/− 0.037)	0.060 (+/− 0.011)
LRC	0.626 (+/− 0.037)	0.649 (+/− 0.033)	**0.657 (+/− 0.061)**	**0.834 (+/− 0.035)**	0.633 (+/− 0.062)	0.603 (+/− 0.122)
MLP	**0.735 (+/− 0.031)**	0.660 (+/− 0.022)	0.644 (+/− 0.057)	0.757 (+/− 0.009)	**0.955 (+/− 0.041)**	0.054 (+/− 0.030)
XGB	0.682 (+/− 0.028)	**0.676 (+/− 0.028)**	0.599 (+/− 0.048)	0.781 (+/− 0.019)	0.806 (+/− 0.031)	0.299 (+/− 0.076)

Decision Tree (DT), Random Forest (RFC), Support Vector Machine (SVM), Complement Naive Bayes (CNB), k-nearest neighbors (KNC), Logistic Regression (LRC), Multi-layer Perceptron (MLP), Extreme Gradient Boosting (XGB)

Highest values are in bold.

For the *Non-permanent Tinnitus Users* plus balanced approach (see Table 4), no new findings can be made, except that the prediction rates are generally somewhat lower.

**Table 4:** Results for *Non-permanent Tinnitus Users*, including standard deviation.

	Non-permanent Tinnitus Users					
	Accuracy	F1-score	AUC	Precision	Sensitivity	Specificity
DT	0.598 (+/− 0.045)	0.611 (+/− 0.036)	0.522 (+/− 0.034)	0.753 (+/− 0.018)	0.681 (+/− 0.067)	0.359 (+/− 0.052)
RFC	0.641 (+/− 0.052)	0.636 (+/− 0.040)	0.554 (+/− 0.076)	0.752 (+/− 0.021)	0.769 (+/− 0.076)	0.273 (+/− 0.063)
SVM	0.554 (+/− 0.084)	0.576 (+/− 0.085)	0.578 (+/− 0.086)	**0.789 (+/− 0.056)**	0.539 (+/− 0.119)	0.595 (+/− 0.111)
CNB	0.519 (+/− 0.107)	0.538 (+/− 0.112)	0.580 (+/− 0.107)	0.777 (+/− 0.087)	0.484 (+/− 0.154)	**0.622 (+/− 0.132)**
KNC	0.702 (+/− 0.045)	0.641 (+/− 0.033)	0.576 (+/− 0.077)	0.743 (+/− 0.017)	0.914 (+/− 0.067)	0.093 (+/− 0.070)
LRC	0.529 (+/− 0.097)	0.550 (+/− 0.097)	0.572 (+/− 0.104)	0.779 (+/− 0.076)	0.505 (+/− 0.139)	0.597 (+/− 0.132)
MLP	**0.720 (+/− 0.036)**	**0.648 (+/− 0.024)**	**0.601 (+/− 0.092)**	0.746 (+/− 0.011)	**0.943 (+/− 0.057)**	0.078 (+/− 0.055)
XGB	0.632 (+/− 0.061)	0.635 (+/− 0.048)	0.576 (+/− 0.088)	0.761 (+/− 0.027)	0.734 (+/− 0.094)	0.339 (+/− 0.096)

Decision Tree (DT), Random Forest (RFC), Support Vector Machine (SVM), Complement Naive Bayes (CNB), k-nearest neighbors (KNC), Logistic Regression (LRC), Multi-layer Perceptron (MLP), Extreme Gradient Boosting (XGB)

Highest values are in bold.

For the *Rather absent Tinnitus Users* plus balanced approach (see Table 5), the results are even lower than for *Non-permanent Tinnitus Users*. This indicates above all that the tinnitus and non-tinnitus classes are too poorly distributed in question 1 of the EMA-D questionnaire. Furthermore, as we analyze specific subsets of users, we would have expected higher results compared to non-permanent Tinnitus Users.

**Table 5:** Results for *Rather absent Tinnitus Users*, including standard deviation.

	Rather absent Tinnitus Users					
	Accuracy	F1-score	AUC	Precision	Sensitivity	Specificity
DT	0.565 (+/− 0.045)	0.573 (+/− 0.038)	0.518 (+/− 0.030)	0.702 (+/− 0.020)	0.643 (+/− 0.076)	0.389 (+/− 0.056)
RFC	0.599 (+/− 0.049)	0.593 (+/− 0.037)	0.548 (+/− 0.075)	0.704 (+/− 0.020)	0.723 (+/− 0.084)	0.319 (+/− 0.062)
SVM	0.566 (+/− 0.095)	0.578 (+/− 0.096)	0.603 (+/− 0.111)	**0.765 (+/− 0.061)**	0.531 (+/− 0.135)	**0.646 (+/− 0.094)**
CNB	0.531 (+/− 0.090)	0.541 (+/− 0.090)	0.586 (+/− 0.100)	0.741 (+/− 0.074)	0.494 (+/− 0.141)	0.614 (+/− 0.136)
KNC	0.644 (+/− 0.052)	**0.607 (+/− 0.038)**	0.586 (+/− 0.087)	0.703 (+/− 0.022)	0.839 (+/− 0.094)	0.205 (+/− 0.091)
LRC	0.548 (+/− 0.100)	0.558 (+/− 0.102)	0.583 (+/− 0.088)	0.733 (+/− 0.077)	0.534 (+/− 0.152)	0.578 (+/− 0.123)
MLP	**0.663 (+/− 0.039)**	0.606 (+/− 0.033)	**0.609 (+/− 0.100)**	0.702 (+/− 0.017)	**0.891 (+/− 0.069)**	0.149 (+/− 0.075)
XGB	0.598 (+/− 0.055)	0.599 (+/− 0.044)	0.573 (+/− 0.082)	0.716 (+/− 0.021)	0.693 (+/− 0.101)	0.384 (+/− 0.080)

Decision Tree (DT), Random Forest (RFC), Support Vector Machine (SVM), Complement Naive Bayes (CNB), k-nearest neighbors (KNC), Logistic Regression (LRC), Multi-layer Perceptron (MLP), Extreme Gradient Boosting (XGB)

Highest values are in bold.

**Figure 1:**
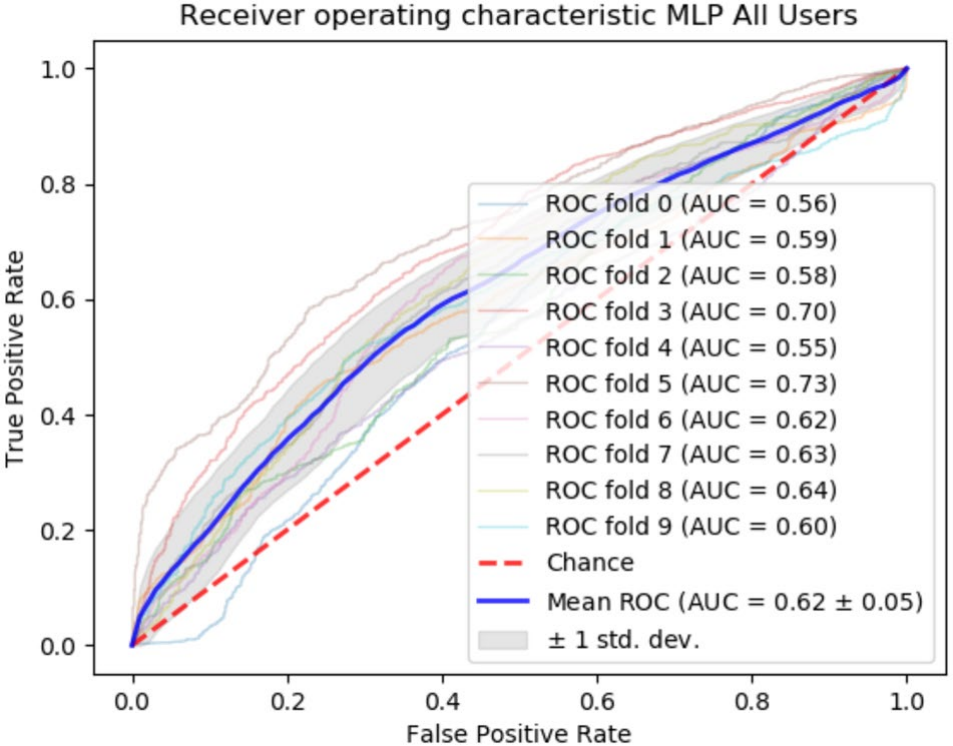
10-folds ROC curve, AUC MLP.

Across all datasets, the multi-layer perceptron classifier (i.e., neural network) is the approach with the best accuracy. The 10-fold cross validated AUC of the MLP classifier for all users is depicted in Fig.  1. However, other approaches (e.g., KNC, XGB, or RFC) also provide promising results. Illustrations of all other approaches can be found in the supplementary material.

### Results downsampling

In the case of downsampling for the five user groups, it is noticeable that the prediction rates decrease and that in some cases other classifiers only perform better than when balanced. This suggests that imputation can either be improved or is even counterproductive in this case. However, it could also indicate that very meaningful values were eliminated during downsampling.

When all users are considered (see Table 6), the results do not vary much between classifiers, but for the first time, the RFC classifier is stronger than before.

**Table 6:** Results for all users (downsampled), including standard deviation.

	All Users down					
	Accuracy	F1-score	AUC	Precision	Sensitivity	Specificity
DT	0.643 (+/− 0.012)	0.645 (+/− 0.015)	0.648 (+/− 0.013)	0.641 (+/− 0.012)	0.651 (+/− 0.026)	0.635 (+/− 0.023)
RFC	0.665 (+/− 0.013)	**0.668 (+/− 0.013)**	0.731 (+/− 0.013)	0.662 (+/− 0.014)	**0.674 (+/− 0.014)**	0.655 (+/− 0.017)
SVM	0.618 (+/− 0.007)	0.604 (+/− 0.007)	0.663 (+/− 0.009)	0.627 (+/− 0.008)	0.582 (+/− 0.011)	0.653 (+/− 0.014)
CNB	0.580 (+/− 0.006)	0.539 (+/− 0.007)	0.635 (+/− 0.006)	0.598 (+/− 0.008)	0.490 (+/− 0.009)	0.671 (+/− 0.013)
KNC	0.646 (+/− 0.013)	0.646 (+/− 0.014)	0.698 (+/− 0.013)	0.646 (+/− 0.013)	0.645 (+/− 0.015)	0.646 (+/− 0.014)
LRC	0.607 (+/− 0.006)	0.589 (+/− 0.009)	0.650 (+/− 0.008)	0.616 (+/− 0.007)	0.564 (+/− 0.015)	0.649 (+/− 0.013)
MLP	0.644 (+/− 0.010)	0.631 (+/− 0.014)	0.700 (+/− 0.010)	0.656 (+/− 0.013)	0.608 (+/− 0.026)	0.680 (+/− 0.026)
XGB	**0.667 (+/− 0.011)**	0.658 (+/− 0.011)	**0.732 (+/− 0.009)**	**0.676 (+/− 0.013)**	0.642 (+/− 0.011)	**0.692 (+/− 0.017)**

Decision Tree (DT), Random Forest (RFC), Support Vector Machine (SVM), Complement Naive Bayes (CNB), k-nearest neighbors (KNC), Logistic Regression (LRC), Multi-layer Perceptron (MLP), Extreme Gradient Boosting (XGB)

Highest values are in bold.

For *Power Users*, the picture is similar (see Table 7). However, compared to the balanced approach, the general jump between all users and *Power Users* is larger this time.

**Table 7:** Results for *Power Users* (downsampled), including standard deviation.

	Power Users down					
	Accuracy	F1-score	AUC	Precision	Sensitivity	Specificity
DT	0.734 (+/− 0.011)	0.738 (+/− 0.009)	0.739 (+/− 0.011)	0.729 (+/− 0.018)	0.748 (+/− 0.018)	0.720 (+/− 0.030)
RFC	0.773 (+/− 0.013)	**0.773 (+/− 0.013)**	0.855 (+/− 0.011)	0.775 (+/− 0.017)	**0.771 (+/− 0.021)**	0.775 (+/− 0.023)
SVM	0.684 (+/− 0.018)	0.685 (+/− 0.022)	0.722 (+/− 0.017)	0.682 (+/− 0.018)	0.689 (+/− 0.035)	0.679 (+/− 0.026)
CNB	0.563 (+/− 0.018)	0.536 (+/− 0.019)	0.622 (+/− 0.021)	0.572 (+/− 0.023)	0.504 (+/− 0.023)	0.621 (+/− 0.033)
KNC	0.760 (+/− 0.014)	0.757 (+/− 0.017)	0.827 (+/− 0.014)	0.766 (+/− 0.013)	0.749 (+/− 0.030)	0.771 (+/− 0.020)
LRC	0.608 (+/− 0.016)	0.566 (+/− 0.018)	0.665 (+/− 0.015)	0.635 (+/− 0.022)	0.511 (+/− 0.022)	0.706 (+/− 0.028)
MLP	0.730 (+/− 0.011)	0.725 (+/− 0.016)	0.797 (+/− 0.015)	0.739 (+/− 0.023)	0.715 (+/− 0.042)	0.745 (+/− 0.042)
XGB	**0.777 (+/− 0.018)**	0.772 (+/− 0.021)	**0.857 (+/− 0.013)**	**0.788 (+/− 0.015)**	0.757 (+/− 0.034)	**0.796 (+/− 0.017)**

Decision Tree (DT), Random Forest (RFC), Support Vector Machine (SVM), Complement Naive Bayes (CNB), k-nearest neighbors (KNC), Logistic Regression (LRC), Multi-layer Perceptron (MLP), Extreme Gradient Boosting (XGB)

Highest values are in bold.

No further insights are gained from the *Normal Users* (see Table 8). Only, as with the balanced approach, the same classifier beats all others in all metrics, except for sensitivity.

**Table 8:** Results for *Normal Users* (downsampled), including standard deviation.

	Normal Users down					
	Accuracy	F1-score	AUC	Precision	Sensitivity	Specificity
DT	0.633 (+/− 0.010)	0.636 (+/− 0.010)	0.638 (+/− 0.011)	0.630 (+/− 0.011)	0.642 (+/− 0.013)	0.623 (+/− 0.016)
RFC	**0.662 (+/− 0.010)**	**0.668 (+/− 0.010)**	**0.723 (+/− 0.009)**	**0.656 (+/− 0.012)**	0.681 (+/− 0.014)	0.643 (+/− 0.018)
SVM	0.627 (+/− 0.011)	0.647 (+/− 0.012)	0.669 (+/− 0.011)	0.614 (+/− 0.010)	**0.683 (+/− 0.021)**	0.571 (+/− 0.018)
CNB	0.588 (+/− 0.010)	0.553 (+/− 0.011)	0.636 (+/− 0.010)	0.605 (+/− 0.013)	0.510 (+/− 0.014)	**0.666 (+/− 0.018)**
KNC	0.627 (+/− 0.011)	0.632 (+/− 0.012)	0.670 (+/− 0.010)	0.623 (+/− 0.011)	0.642 (+/− 0.015)	0.611 (+/− 0.014)
LRC	0.629 (+/− 0.010)	0.634 (+/− 0.012)	0.668 (+/− 0.011)	0.627 (+/− 0.010)	0.641 (+/− 0.021)	0.618 (+/− 0.019)
MLP	0.641 (+/− 0.014)	0.640 (+/− 0.015)	0.688 (+/− 0.014)	0.641 (+/− 0.017)	0.639 (+/− 0.025)	0.642 (+/− 0.028)
XGB	0.654 (+/− 0.011)	0.654 (+/− 0.014)	0.712 (+/− 0.011)	0.655 (+/− 0.010)	0.653 (+/− 0.021)	0.656 (+/− 0.013)

Decision Tree (DT), Random Forest (RFC), Support Vector Machine (SVM), Complement Naive Bayes (CNB), k-nearest neighbors (KNC), Logistic Regression (LRC), Multi-layer Perceptron (MLP), Extreme Gradient Boosting (XGB)

Highest values are in bold.

There is no further findings compared with the previous results for *Non-permanent Tinnitus Users*, as shown in Table 9.

**Table 9:** Results for *Non-permanent Tinnitus Users* (downsampled), including standard deviation.

	Non-permanent Tinnitus Users down					
	Accuracy	F1-score	AUC	Precision	Sensitivity	Specificity
DT	0.640 (+/− 0.009)	0.648 (+/− 0.012)	0.641 (+/− 0.012)	0.634 (+/− 0.009)	0.663 (+/− 0.023)	0.618 (+/− 0.020)
RFC	**0.665 (+/− 0.011)**	**0.670 (+/− 0.012)**	**0.733 (+/− 0.013)**	0.661 (+/− 0.011)	**0.678 (+/− 0.017)**	0.652 (+/− 0.015)
SVM	0.608 (+/− 0.010)	0.605 (+/− 0.009)	0.640 (+/− 0.012)	0.610 (+/− 0.012)	0.599 (+/− 0.008)	0.617 (+/− 0.017)
CNB	0.569 (+/− 0.012)	0.542 (+/− 0.011)	0.609 (+/− 0.014)	0.579 (+/− 0.014)	0.510 (+/− 0.010)	0.629 (+/− 0.018)
KNC	0.642 (+/− 0.011)	0.643 (+/− 0.010)	0.690 (+/− 0.011)	0.641 (+/− 0.013)	0.646 (+/− 0.013)	0.638 (+/− 0.020)
LRC	0.593 (+/− 0.010)	0.577 (+/− 0.010)	0.618 (+/− 0.013)	0.600 (+/− 0.011)	0.556 (+/− 0.011)	0.629 (+/− 0.014)
MLP	0.637 (+/− 0.008)	0.630 (+/− 0.013)	0.694 (+/− 0.010)	0.643 (+/− 0.015)	0.620 (+/− 0.033)	0.654 (+/− 0.038)
XGB	**0.665 (+/− 0.012)**	0.657 (+/− 0.015)	0.723 (+/− 0.012)	**0.674 (+/− 0.012)**	0.641 (+/− 0.022)	**0.689 (+/− 0.015)**

Decision Tree (DT), Random Forest (RFC), Support Vector Machine (SVM), Complement Naive Bayes (CNB), k-nearest neighbors (KNC), Logistic Regression (LRC), Multi-layer Perceptron (MLP), Extreme Gradient Boosting (XGB)

Highest values are in bold.

With *Rather absent Tinnitus Users* and downsampling (see Table 10), the results are closer to *Non-permanent Tinnitus Users* than in the balanced case.

**Table 10:** Results for *Rather absent Tinnitus Users* (downsampled), including standard deviation.

	Rather absent Tinnitus Users down					
	Accuracy	F1-score	AUC	Precision	Sensitivity	Specificity
DT	0.657 (+/− 0.006)	0.661 (+/− 0.008)	0.662 (+/− 0.007)	0.653 (+/− 0.008)	0.669 (+/− 0.017)	0.644 (+/− 0.017)
RFC	**0.683 (+/− 0.010)**	**0.687 (+/− 0.011)**	**0.753 (+/− 0.012)**	0.678 (+/− 0.011)	**0.697 (+/− 0.015)**	0.669 (+/− 0.015)
SVM	0.620 (+/− 0.012)	0.590 (+/− 0.013)	0.659 (+/− 0.013)	0.641 (+/− 0.016)	0.547 (+/− 0.014)	0.693 (+/− 0.018)
CNB	0.595 (+/− 0.010)	0.578 (+/− 0.012)	0.616 (+/− 0.012)	0.603 (+/− 0.011)	0.556 (+/− 0.016)	0.635 (+/− 0.017)
KNC	0.651 (+/− 0.010)	0.652 (+/− 0.010)	0.703 (+/− 0.011)	0.650 (+/− 0.011)	0.655 (+/− 0.012)	0.647 (+/− 0.015)
LRC	0.601 (+/− 0.012)	0.605 (+/− 0.013)	0.632 (+/− 0.016)	0.600 (+/− 0.012)	0.610 (+/− 0.016)	0.592 (+/− 0.015)
MLP	0.648 (+/− 0.009)	0.633 (+/− 0.017)	0.704 (+/− 0.011)	0.663 (+/− 0.018)	0.607 (+/− 0.041)	0.690 (+/− 0.043)
XGB	0.672 (+/− 0.010)	0.664 (+/− 0.011)	0.739 (+/− 0.011)	**0.681 (+/− 0.010)**	0.649 (+/− 0.013)	**0.696 (+/− 0.010)**

Decision Tree (DT), Random Forest (RFC), Support Vector Machine (SVM), Complement Naive Bayes (CNB), k-nearest neighbors (KNC), Logistic Regression (LRC), Multi-layer Perceptron (MLP), Extreme Gradient Boosting (XGB)

Highest values are in bold.

The basic prediction rate varies between 60 and 70%. At the subtle level, we find differences that are rather unusual, but can also occur depending on the strategy, so they may not have any medical significance. Also, one would have expected the prediction to perform disproportionately better for *Power Users* than for the other classes. Moreover, it is interesting that the algorithms sometimes predict strikingly differently between the balanced and downsampling variants.

## Discussion

At the beginning of the work, we hypothesized along the five user groups with which significance the presence of a tinnitus perception — asked by question 1 in the EMA-D questionnaire — can be predicted by the other questions of the EMA-D questionnaire. For this purpose, we excluded those questions from the EMA-D questionnaire that showed medical correlation (dependent variables, i.e., questions 2, 3, and 8). Since the same objective is in the room for all five user groups and the results do not differ significantly, we discuss the results in their entirety with regard to the three research questions raised. Basically, it is noticeable that despite the different scenarios, a prediction in the range of 50–70% is always possible, with the prediction tending towards 60–70%. Since question 1 of the EMA-D questionnaire has a binary scale (cf. Table 11; 0: *no tinnitus perceived*, 1: *tinnitus perceived*), the ground truth in this case is 50%. Comparing the results with the ground truth, a significant improvement can be obtained, indicating that the dynamic questions of the EMA questionnaire not directly related to question 1 contain predictive power, again for all user groups. However, the TYT dataset is not a small mHealth dataset, so one would have expected a higher predictive value than 70% fluctuating. Therefore, the result should be considered indicative only, even though the ground truth might be exceeded.

However, it is noticeable that the *Power Users* can only achieve a small improvement in the prediction, especially in the case of the *downsampling* scenario with 77.7%. Comparing this with the best result of all users (66.8%), the difference is not that big. On the one hand, this is unfortunate, because one could have expected a better prediction here, since these users use the system more and probably give honest answers over time. On the other hand, the result is very good, at least with respect to the research questions of this paper, namely that the totality of all measurements already gives a representative picture. What provides another very good statement is the fact that in the case of *downsampling* and *Power Users* the best possible result was obtained. Again, no data was imputed in the case of *downsampling*, so obviously the pure data from users who frequently use the platform is more meaningful at the rating level, which supports the basic tendency of the positive prediction of question 1 of the EMA-D questionnaire.

Across all scenarios and data sets, the classifications score results above the baseline regarding sensitivity. In other words, they are able to predict cases of tinnitus. Regarding specificity, some classifications (i.e., DT, KNC, MLP, RFC, and XGB) yield results below the baseline for the balanced scenario, indicating that the prediction of cases in which no tinnitus was reported might not be ideal. This is, however, not the case for the scenario *downsampling* and may indicate that the downsampling approach should be preferred in case the prediction of no tinnitus cases is relevant.

It can also be seen that the two strategies *balanced* and *downsampling* produce little difference, at least as far as the prediction rates are concerned. The differences in the classifications related to *balanced* and *downsampling* are partly considerable. For example, in the case of *balanced*, the MLP classifier performs best on average, while in the case of *downsampling*, the RFC and XGB classifiers perform better. Since both are tree methods, it is also possible that values are eliminated in *downsampling* or added in *balanced* that influence the respective tree method. This shows nicely how the individual methods react to data changes. Since the basic tendency of the predictions remains the same, it can be assumed that the different performance of the classifiers is due to the *balanced* and *downsampling* strategies and has nothing to do with medical significance. Precisely for the reasons mentioned above, we consider the work as a further contribution in the field of mHealth / Machine Learning / Tinnitus as well as mHealth / Machine Learning in general. When classifying at assessment level, ML-methods seem to perform similarly on tabular mHealth data, again independent of how the dataset is processed in terms of balance as shown in this work. One could also speak here of a comprehensive testing of the mentioned strategies for the assessment level. Since the dependent questions in the EMA-D questionnaire were also excluded, it can be assumed that with more data, tinnitus perception can be predicted even better on the basis of the independent variables used in our EMA-D questionnaire: mood, arousal, stress, concentration. Therefore, a first indicator can be given that the independent variables are able to predict the momentary tinnitus on the assessment level.

In terms of clinical relevance, the outcome of this paper has enabled two main contributions. First, we want to better understand and learn how smartphone apps need to be developed to best support tinnitus patients in particular and patients in general, including in daily data collection. Only in this way, will we be able to collect data in the future that will give us further insight into tinnitus. Identifying subgroups is highly relevant clinically, as there is no general treatment method and thus the hope is to develop specific treatments for subgroups. Findings like this take us further to better address subgroups. For example, as an outcome, we need to identify early on what are power users and what are normal users to incentivize accordingly. Second, the realization has matured that, data-driven, the various alternatives (classifiers, data preparation by the imbalances) related to subgroups do not have as great an impact as might have been suspected. Nevertheless, we will need more power users, because they promise us the deeper results in the future. Thus, it is imperative to find motivational mechanisms that do not have a detrimental effect on the other side (that data are collected only due to the motivational mechanism).

The work has some limitations, which must be considered carefully. First, the prediction is performed on the basis of the assessment level. In other work, it could be shown what a difference this can make with respect to the user level^17,22^. Nevertheless, we are currently comparing the user and assessment level with the various options in a larger study. A further bias may arise from the user interface elements of the independent questions. Sliders bear the risk of the anchor effect^23^ and can thus distort a result^15^, since the independent questions are all slider questions, this has to be taken into account. Furthermore, a selection bias can be assumed, even though many things were compared in Table 12. Since TYT is an open observational study, i.e., we do not hand out a clear study protocol, the participating users can very much decide for themselves how the app is used (i.e., the number, daytime, pattern (random during the day / always at the same time) can be defined individually), so a selection bias is to be expected^24^. Additionally, we do not obtain information about the number of prompts a user is exposed to. Consequently, some individuals may respond to all prompts they are exposed to, whereas others may only respond to few of the EMA prompts. Therefore, we cannot examine how the number of prompts completed is related to the number of prompts to which users were exposed. However, due to the number of EMA responses, it is to be assumed that power users respond more frequently to prompts compared to normal users. Since Android and iOS never look 100% the same in terms of user interface, information bias must also be assumed, since TYT is available on both platforms. In principle, the two strategies *balanced* and *downsampling* can also be seen as bias, but since the results do not vary greatly, this bias can be seen as smaller. However, the fact that the users answered very strongly in the two classes whether tinnitus is perceived or not, this basic circumstance must be seen as bias. We do not know whether the classifiers could distinguishably see enough perceived and not perceived tinnitus states to an individual. Another limitation arises from the different user groups. In this case both tests for dependent as well as independent samples are not 100% correct, because there are partial dependencies and overlaps between the user groups. However, to ensure comparability between the user groups, we conducted tests for independent samples.

We view the analyses performed as another indicator that machine learning in mHealth data presents many challenges that must be approached with caution. From the data collection side (information bias, e.g., slider or Android vs. iOS) to the nature of the mHealth study (selection bias, e.g., for what reasons do I answer), there is much to consider that is not related to the actual machine learning. There is also the issue of assessment and user level, and how to validate to get more robust results. In this tension, it seems interesting for TYT to note that at the assessment level, the different strategies followed do not have as much impact as one might have suspected. It can be concluded that the as-is data with good cross-validation leads to meaningful results, at least in the sense of what the data basically give. The next point that should definitely be explored is how user and assessment levels differ.

## Materials and methods

### Overview

### Data source

The TYT mHealth platform has been in operation since 2014 and has been continuously evolved since then. The platform consists of a registration and information website (https://www.trackyourtinnitus.org/), a native mobile application available for both iOS and Android, and a central backend that stores the collected data in a relational database. The mobile applications (iOS, Android) track users’ individual tinnitus and tinnitus-related variables by asking them to complete EMA assessment questionnaires at randomly selected times of day (so-called EMA-D questionnaires). The structure of the EMA questionnaire is shown in Table 11. The exact procedure of the TYT application has been described in previous work^4,5^.

Tinnitus is the perception of an internal sound in the ears with no corresponding external sound. Symptoms have been found to be subjective and to vary over time. Therefore, TYT was developed to assess this individual variability of symptoms based on EMA and MCS^3^. TYT uses self-report questionnaires as a data collection tool to collect data on the user’s individual tinnitus. The collected responses, in turn, are stored in the central relational database along with a set of metadata (e.g., timestamp and user agent). Details of the TYT dataset as well as the underlying database structure have also been described in previous work^24^.

**Table 11:** Questions of the EMA-D questionnaire in the TYT smartphone application, along with their scale and the dimension that is measured^3,5^.

#	Question	Scale	Dimension
1	Did you perceive the tinnitus right now?	BS	Perception
2	How loud is the tinnitus right now?	VAS	Loudness
3	How stressful is the tinnitus right now?	VAS	Distress
4	How is your mood right now?	VAS	Mood
5	How is your arousal right now?	VAS	Arousal
6	Do you feel stressed right now?	VAS	Stress
7	How much did you concentrate on the thingsyou are doing right now?	VAS	Concentration
8	Do you feel<reported worst symptom> right now?	BS	Worst symptom

BS: Binary Scale, VAS: Visual Analogue Scale.

### User statistics

To ensure comparability between user groups, we examined important clinical baseline characteristics of age distribution, handedness, and family history of tinnitus. For age distribution, there were no statistically significant differences between group means, as revealed by a one-way ANOVA ($F(4, 1621) = 1.81, p=0.12$). For handedness, a $\chi ^2$-independence test showed that there was no significant relationship between user groups, $\chi ^2(8, N=1654) = 0.91, p=1.0$. We obtained the same result for tinnitus family history, $\chi ^2((4, N=1650) = 0.16, p=1.0$. The different degrees of freedom resulted from missing values for some users. A summary of baseline characteristics for the five user groups is provided in Table 12.

**Table 12:** Statistical comparison of the five user groups. $\chi ^2$ tests for *handedness* ($\chi ^2(8, N=1654) = 0.91, p=1.0$) and *family history of tinnitus complaints* ($\chi ^2((4, N=1650) = 0.16, p=1.0$) suggest that there are no significant differences between the groups. The same result appears for the *age* distributions.

n	n_users	All	Power	Normal	Non-permanent	Rather absent
		518	22	496	361	280
Age	count	500	21	480	352	273
	mean	1969.72	1964.62	1969.9	1970.93	1971.35
	std	13.57	10.39	13.67	13.98	13.78
	min	1935	1940	1935	1935	1935
	25%	1960	1958	1960	1961	1962
	50%	1969	1966	1969	1970	1971
	75%	1979	1974	1979.25	1980	1981
	max	2019	1981	2019	2019	2018
Handedness	Right	76.30%	72.70%	76.50%	75.90%	75.70%
	Both Sides	12.90%	13.60%	13.10%	12.30%	12.30%
	Left	10.80%	13.60%	10.40%	11.80%	12.00%
Family history of tinnitus complaints	No	76.40%	77.30%	76.40%	75.60%	76.70%
	Yes	23.60%	22.70%	23.60%	24.40%	23.30%

### Data preparation

To answer research questions 1–3 and understand the machine learning analysis, Fig. 3 illustrates the steps taken during our data preparation. For the machine learning analysis, we considered 5 datasets, each corresponding to a research question. According to question 1, we analyzed 45,935 answers to the harmonized EMA-D questionnaire from all users (DS1). To further validate the results of the machine learning analysis, and to obtain different insights into the presence or absence of tinnitus, and also to answer research questions 2 & 3, we considered four additional subsets. *Power Users* (DS2) are users with 400 or more EMA questionnaire answers, i.e., the users in the top 5% quantile regarding the number of completed EMA questionnaires. They use the platform excessively and we assume that their subset therefore contains insightful knowledge, forming the first sample to answer RQ2. *Normal Users* (DS3) have completed less than 400 EMA questionnaires. Consequently, they correspond to the other 95% of users forming the second sample for RQ2. Since they are the opposite of *Power Users*, we hope to find new correlations in this subset that are unrelated to the number of questionnaires answered. While some users reported either always or never having tinnitus (e.g., all of their EMA questionnaires contain either only 1 or 0 for question 1), we considered this scenario with the remaining two subsets. Users with non-permanent tinnitus (DS4) reported both the presence and absence of tinnitus and at least once, whereas users with rather absent tinnitus (DS5) reported the absence of tinnitus at least three times and the presence at least once in all questionnaires they answered. These two datasets refer to RQ3.

This leads us to 5 different sub-datasets (DS) of our dataset:

**DS1:** All Users
  This dataset contains all answered EMA questionnaires of users with at least 11 filled out questionnaires and no missing values. It contains 45,935 filled-out questionnaires from 518 different users.
**DS2:** Power Users
  This data set contains all answered EMA questionnaires of users with 400 or more filled-out questionnaires. It contains 14,743 questionnaire answers from 22 different users.
**DS3:** Normal Users
  This data set consists of 31,192 answered EMA questionnaires from 496 users with less than 400 filled-out questionnaires.
**DS4:** Non-permanent Tinnitus Users
  This data set represents all users that reported both presence and absence of tinnitus at least once. This leads to 34,998 questionnaire answers from 361 different users.
**DS5:** Rather absent Tinnitus Users
  As a subset of DS4, this data set corresponds to all users that reported the absence of tinnitus at least 3 times and the presence at least once. 28,964 questionnaires from 280 users are part of this data set.

**Table 13:** Additional possible thresholds for *Power User*.

		#Questionnaires	%	#User	%
400	Power users	14743	32.10	22	4.25
	Normal users	31192	67.90	496	95.75
200	Power users	22745	49.52	51	9.85
	Normal users	23190	50.48	467	90.15
600	Power users	8406	18.30	9	1.74
	Normal users	37529	81.70	509	98.26

**Figure 2:**
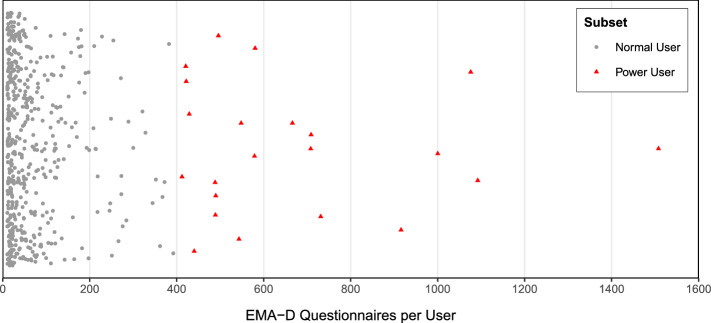
Number of EMA-D questionnaires per unique user. Users with less than 11 submitted questionnaires are omitted. *Normal Users* submitted 11–399 questionnaires and *Power Users* 400 or more questionnaires.

Of course, there are also other possible thresholds than 400 questionnaires per user to distinguish between *Power Users* and *Normal Users* (see RQ2). Alternatives can be either more restrictive, such as 600 questionnaires per user or less restrictive such as 200 questionnaires. However, the setting of this threshold affects the size of the underlying data set both in terms of number of users and questionnaires (see Table 13). On the one hand, setting the threshold to 200 questionnaires per *Power User* results in 51 users (9.85%) and 22745 (49.52%) questionnaires. Using the top $\sim$ 10% of users seems to be a solid estimate; however, it would also result in $\sim$ 50% of questionnaires. On the other hand, setting the threshold to 600 questionnaires per *Power User* would result in 9 users (1.74%) and 8406 (18.30%) questionnaires. In this scenario, the proportion of users would be too low, and considering only 18.30% of questionnaires seems too restrictive. Therefore, we set the threshold to 400, resulting in 4.25% of users and 32.1% of questionnaires. The threshold of 400 is close to the 95th percentile and also includes  30% of all questionnaires. Consequently, all values are a good average between 200 and 600. In addition, Fig. 2 shows the number of questionnaires per user. A separation at 400 questionnaires can be seen, which also supports the decision.

**Figure 3:**
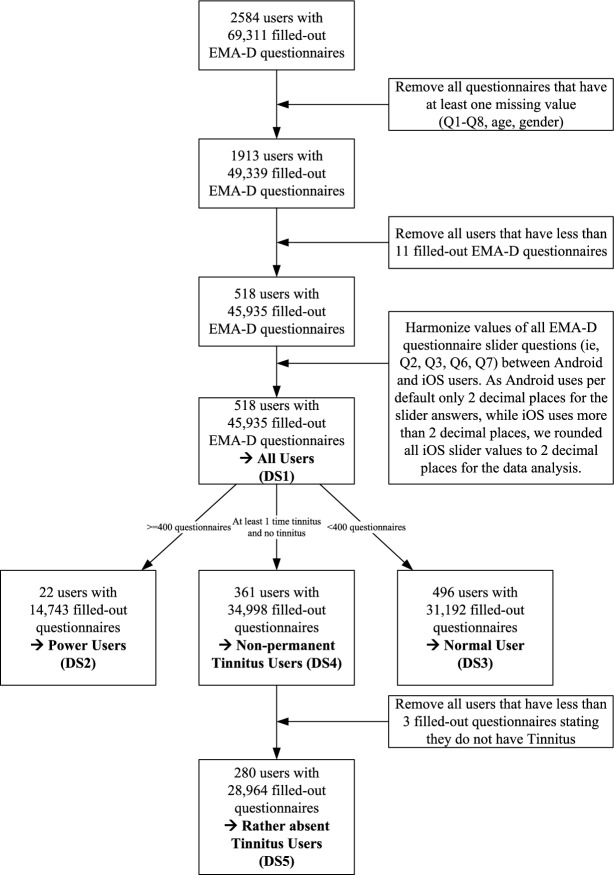
Data Preparation process.

**Figure 4:**
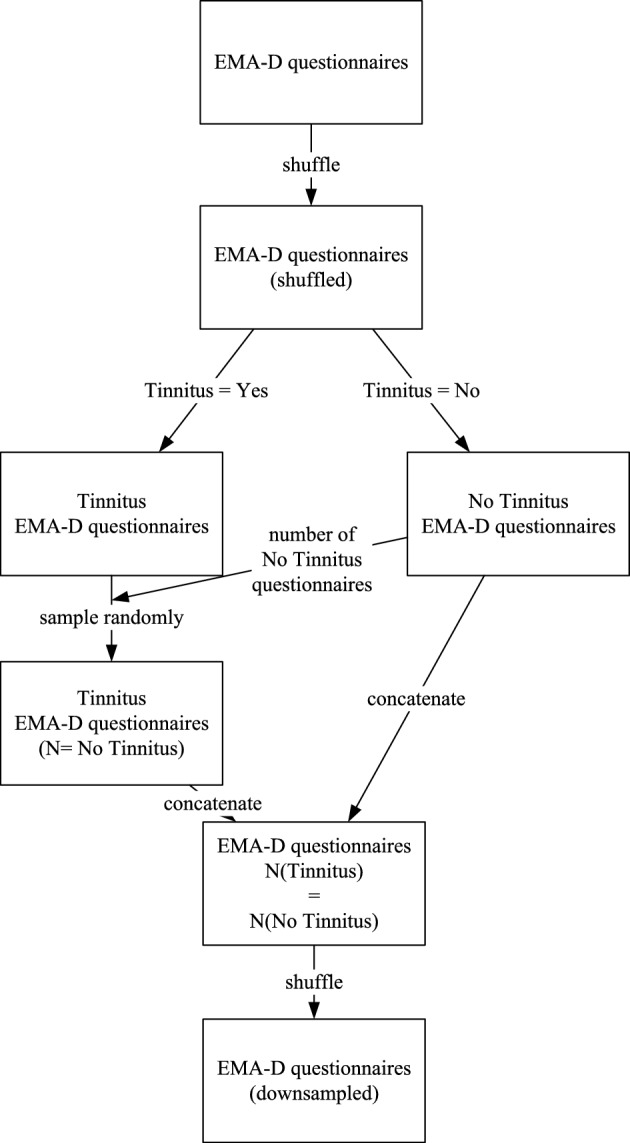
Downsampling process.

### Machine learning analysis

We applied eight different machine learning approaches with the goal of predicting the presence or absence of tinnitus given an assessment in the context of the EMA-D data. In our dataset, this corresponds to the answer to question 1. The following eight approaches were applied to the different datasets: a Decision Tree (DT), a Random Forest Classifier (RFC), a Support Vector Machine (SVM), a complementary Naive Bayes classifier (CNB), a K-nearest Neighbors Classifier (KNC), a Logistic Regression Classifier (LRC), a Multi-layer Perceptron Classifier (MLP), and an Extreme Gradient Boosting Classifier (XGB). These approaches were selected because they represent a variety of different classification approaches. It should be noted that all machine learning approaches were applied at the assessment level of the EMA-D questionnaires. In other words, a user’s score may be included in both the training and validation datasets, which could introduce bias. An alternative approach would be to separate users included in either the training or validation dataset. However, if users are only part of the training or validation dataset, it is important to ensure that there are no user characteristics between the two groups of users that could lead to bias. The TYT application follows an EMA-driven approach where random, voluntary, and dynamic assessments are the main goal. As a result, it is difficult to identify sufficiently large groups of users with similar rating characteristics. Other possibilities in this context are stratified cross validation^25^, external validation^26^ or LOSO^27^. In further experiments, these methods will be applied to TYT data and also compared with other mHealth data sources. Beyond these considerations, the dataset was prepared as follows and before training the classifiers: In the case of *downsampling*, as many entries were randomly eliminated from the majority class (i.e., entries that report tinnitus), so that there were the same number of entries as in the minority class (i.e., entries that do not report tinnitus). This approach is called downsampling. The concrete steps for the downsampling approach are depicted in Fig. 4. Another approach, which we call *balanced*, adapts the weights of classes inversely proportional to class frequencies in the input data, if possible. This was the case for DT, RFC, SVM, XGB, and LR. Remaining approaches do not allow for weight adjustments and were therefore used with default specifications.

Finally, it should be mentioned here once again that the classifications were carried out separately on the basis of the five user groups shown above. The groups relativize the assessment-level bias, but in order to be comparable, all 5 groups were calculated at the assessment level.

Furthermore, for the KNC, we calculated the mean error for all k values between 1 and 500 and selected the k value with the lowest mean error for each data set. In addition, there is a intuitive relationship between question 1, question 2, and question 3 (see Table 11). Question 8 is dynamic in the sense that the question may vary from user to user depending on the worst symptom collected in a previous questionnaire. Therefore, including questions 2, 3, and 8 in models predicting question 1 would introduce bias. To avoid this bias, we exclude questions 2, 3, and 8 from the machine learning analysis. In short, we predict the answer to question 1 based solely on the answers to questions 4, 5, 6, and 7.

To further validate our results, each reported result is based on a 10-fold cross-validation. In this process, the entire data set was divided into 10 equal parts. Nine of the ten parts were used to train the model, while the remaining part was used for testing and optimization. This is then repeated 10 times, whereas each repetition uses a different part of the data set for testing and the remaining 9 parts for training. To address the imbalance in our data, we split the data using a stratified strategy. This ensures that both classes are correctly represented in both the training and testing sets. For classifiers that support this option, we also adjusted the weights of the two classes to account for the imbalance. By adjusting the weights to be inversely proportional to the class frequency, we ensure that the classifiers do not ignore any classes. The whole procedure was repeated 10 times and the averages were calculated over all 10 runs. To corroborate our results, we applied the above procedure to all 5 data sets shown in Fig. 3, and on each of the eight machine learning approaches.

Six different metrics are used to evaluate the results: Accuracy, the weighted F1 score, the area under the Receiver Operating Characteristic Curve (AUC), Precision, Sensitivity, and Specificity. All analyses were performed in the following environment: a laptop with an i7 core (2.60 GHz) and Python scikit-Learn.

### Ethics approval

The study was approved by the Ethics Committee of the University Clinic of Regensburg (ethical approval No. 15-101-0204).

### Informed Consent

All users read and approved the informed consent before participating in the study.

## Supplementary Information

Supplementary Information.

## Data Availability

The data presented in this study are available on request from the corresponding author. The data are not publicly available due to privacy reasons.
