# Supplementary material for: Predicting the presence of tinnitus using ecological momentary assessments
Source: Sci Rep. 2023 Jun 2;13:8989. doi: 10.1038/s41598-023-36172-7 (PMC10238428; doi:10.1038/s41598-023-36172-7)
Supplement: Supplementary file 1 — Supplementary Information. [file 41598_2023_36172_MOESM1_ESM.pdf]

Predicting the Presence of Tinnitus using Ecological Momentary Assessments

Marius Breitmayer[1],*, Michael Stach[2], Robin Kraft[1,3], Johannes Allgaier[2], Manfred Reichert[1], Winfried Schlee[4,5], Thomas Probst[6], Berthold Langguth[5], and Rüdiger Pryss[2]
[1]Institute of Databases and Information Systems, Ulm University, Ulm, Germany
[2]Institute of Clinical Epidemiology and Biometry, University of W¨urzburg, W¨urzburg, Germany
[3]Department of Clinical Psychology and Psychotherapy, Ulm University, Ulm, Germany
[4]Institute for Information and Process Management, Eastern Switzerland University of Applied Sciences, St. Gallen, Switzerland
[5]Clinic and Policlinic for Psychiatry and Psychotherapy, University of Regensburg, Regensburg, Germany
[6]Department for Psychotherapy and Biopsychosocial Health, Danube University Krems, Austria
*marius.breitmayer@uni-ulm.de

Supplementary Material

Code Availability

The source code for this manuscript is available on request from the corresponding author.

Additional 10-fold ROC curves

Decision Tree (DT)

All Users:

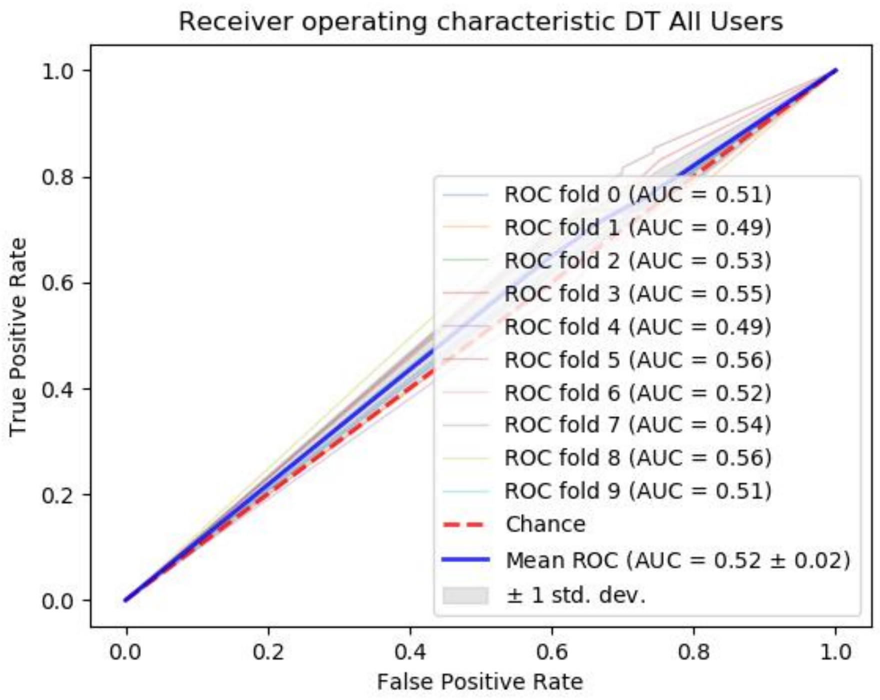

Supplementary Figure S1 10-folds ROC curve, AUC DT, All Users

Power Users:

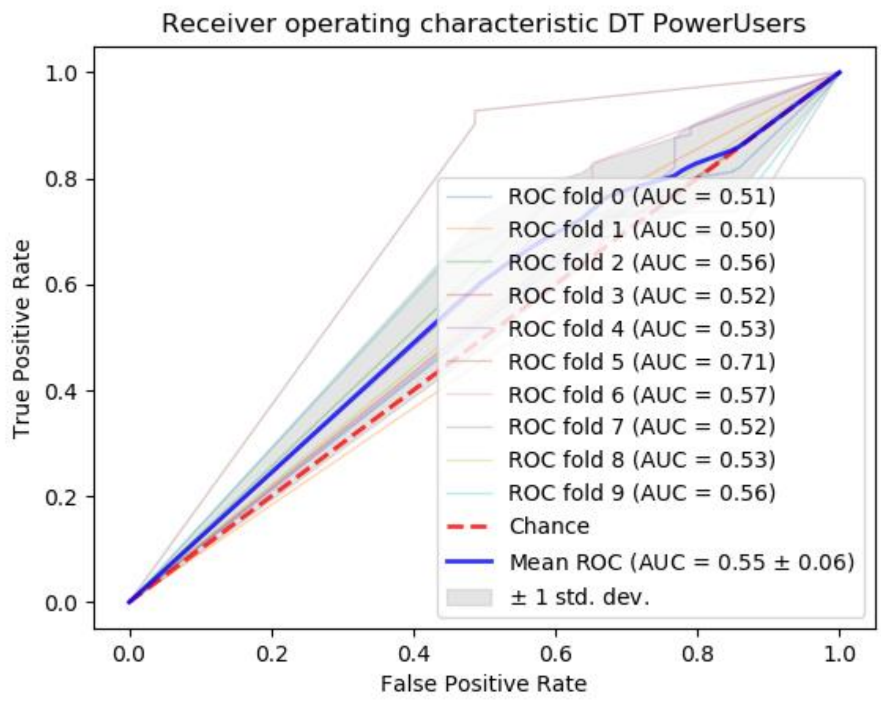

Supplementary Figure S2 10-folds ROC curve, AUC DT, Power Users

Normal Users:

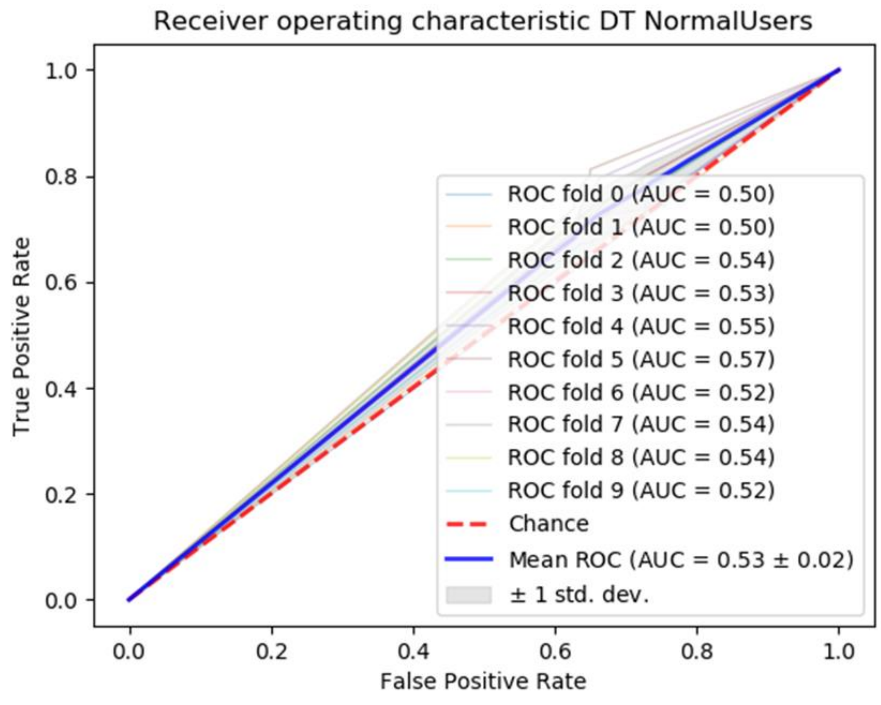

Supplementary Figure S3 10-folds ROC curve, AUC DT, Normal Users

Non-permanent Tinnitus Users:

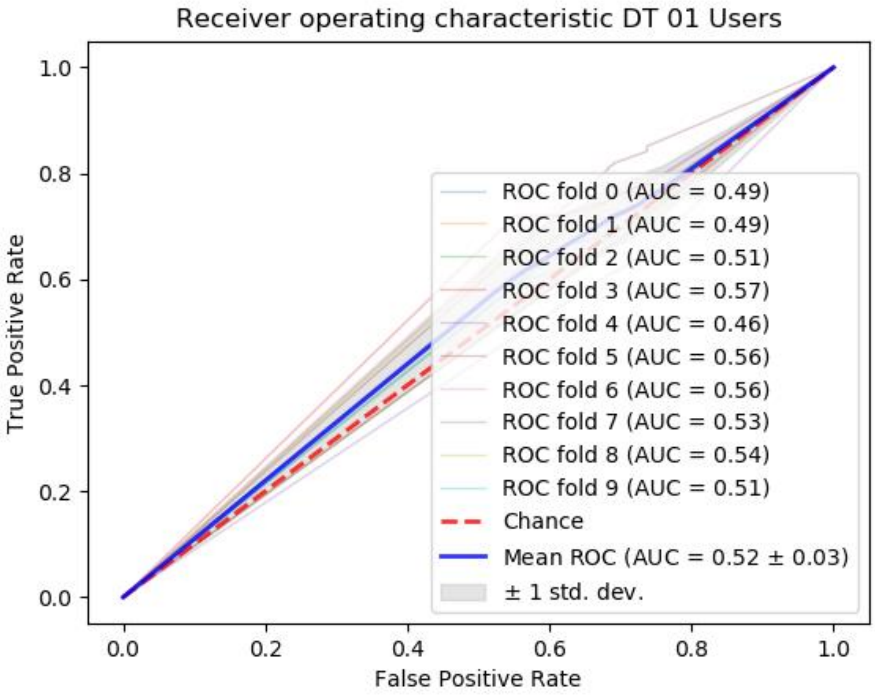

Supplementary Figure S4 10-folds ROC curve, AUC DT, Non-permanent Tinnitus Users

Rather absent Tinnitus Users:

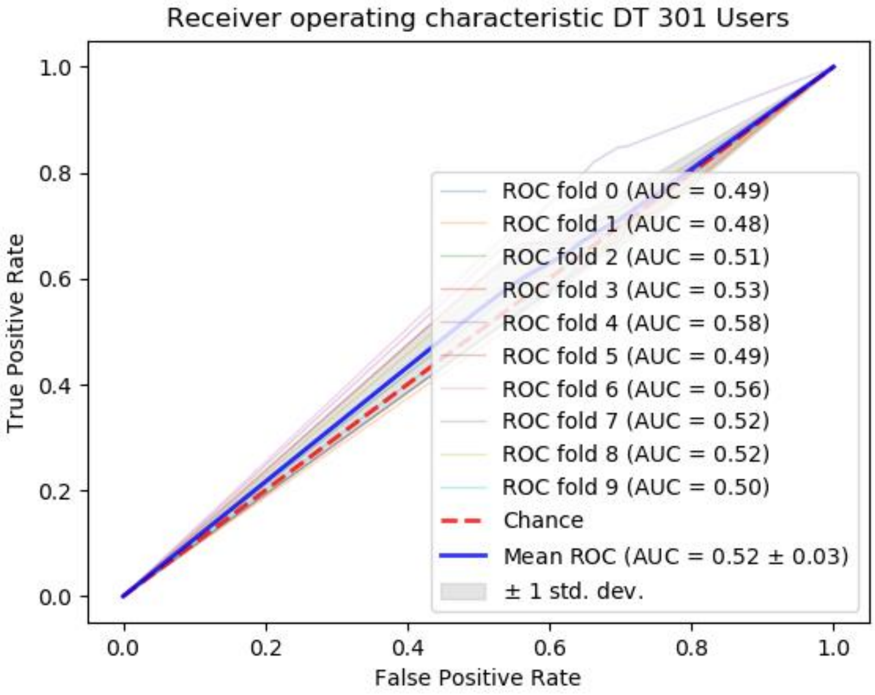

Supplementary Figure S5 10-folds ROC curve, AUC DT, Rather absent Tinnitus Users

Random Forest (RFC)

All Users:

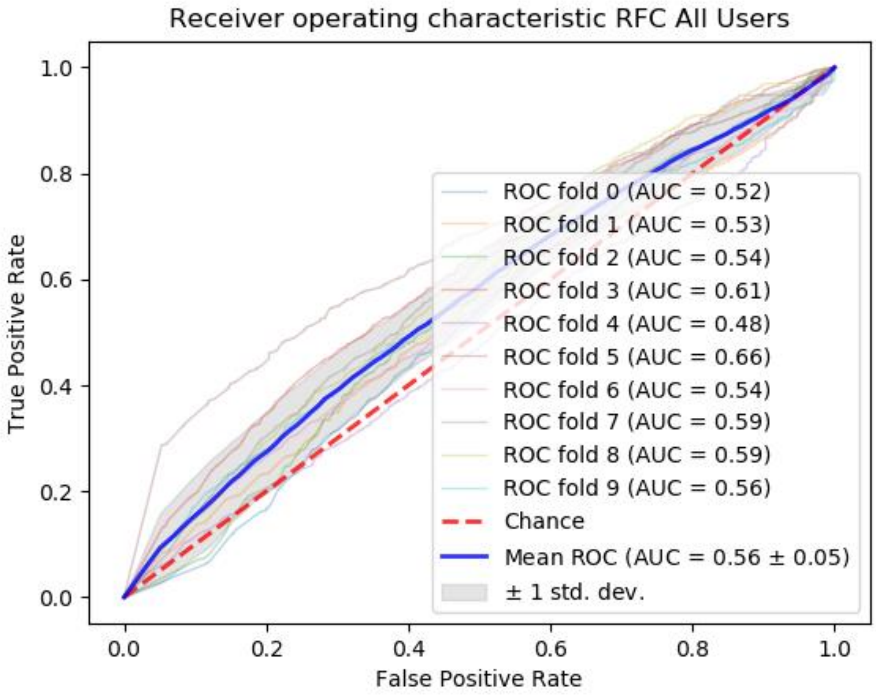

Supplementary Figure S6 10-folds ROC curve, AUC RFC, All Users

Power Users:

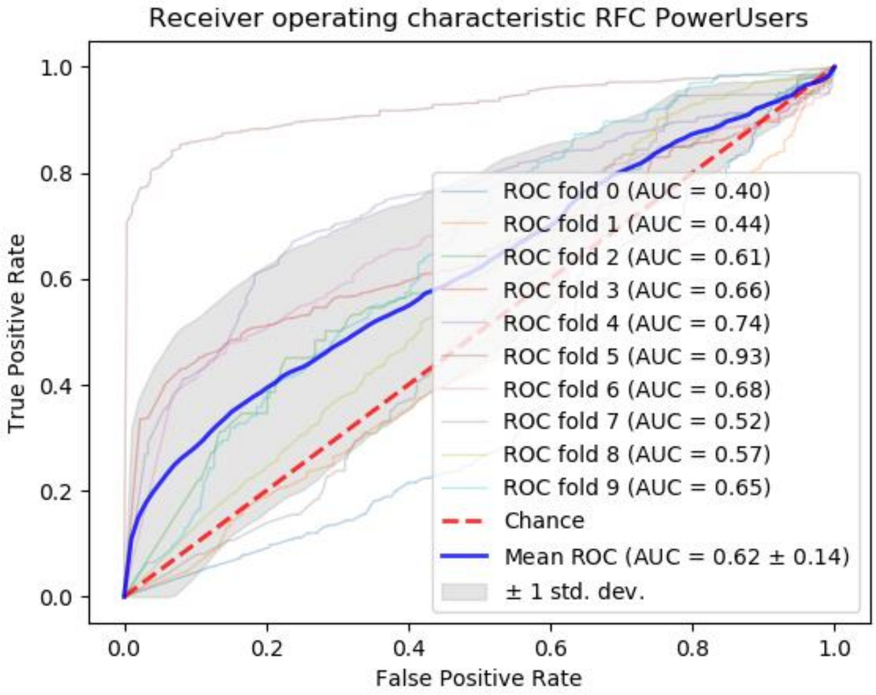

Supplementary Figure S7 10-folds ROC curve, AUC RFC, Power Users

Normal Users:

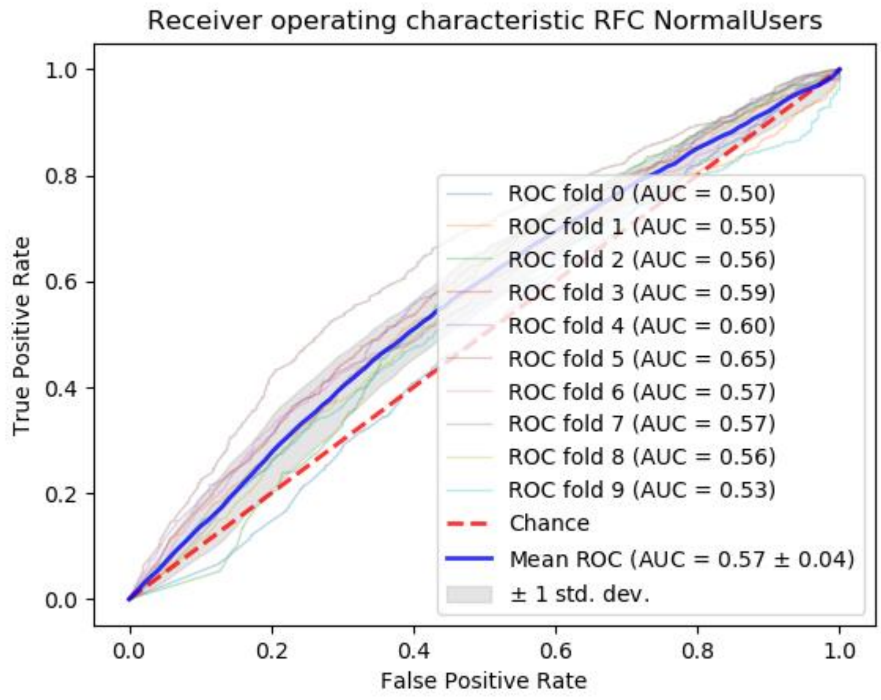

Supplementary Figure S8 10-folds ROC curve, AUC RFC, Normal Users

Non-permanent Tinnitus Users:

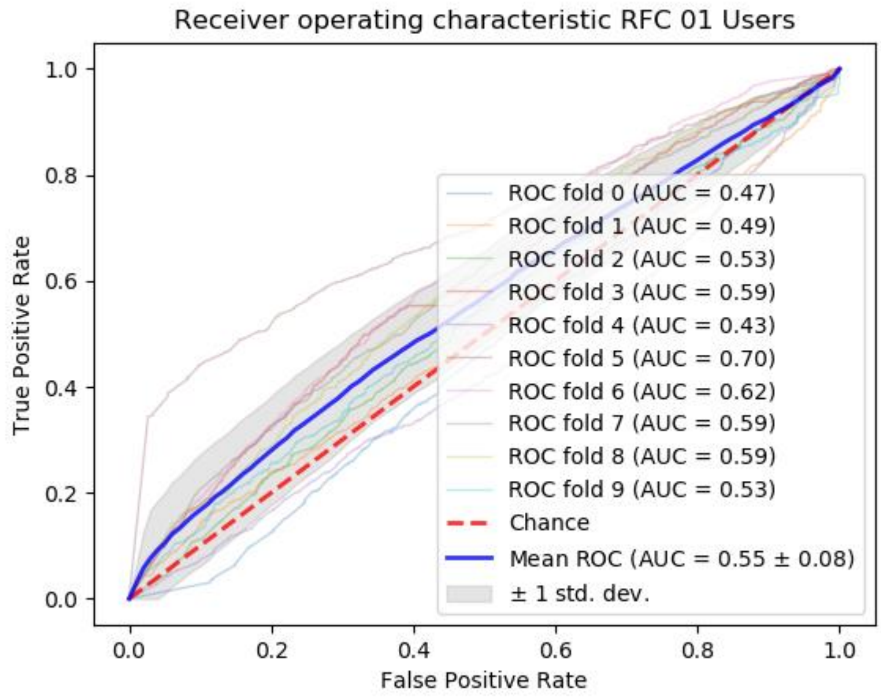

Supplementary Figure S9 10-folds ROC curve, AUC RFC, Non-permanent Tinnitus Users

Rather absent Tinnitus Users:

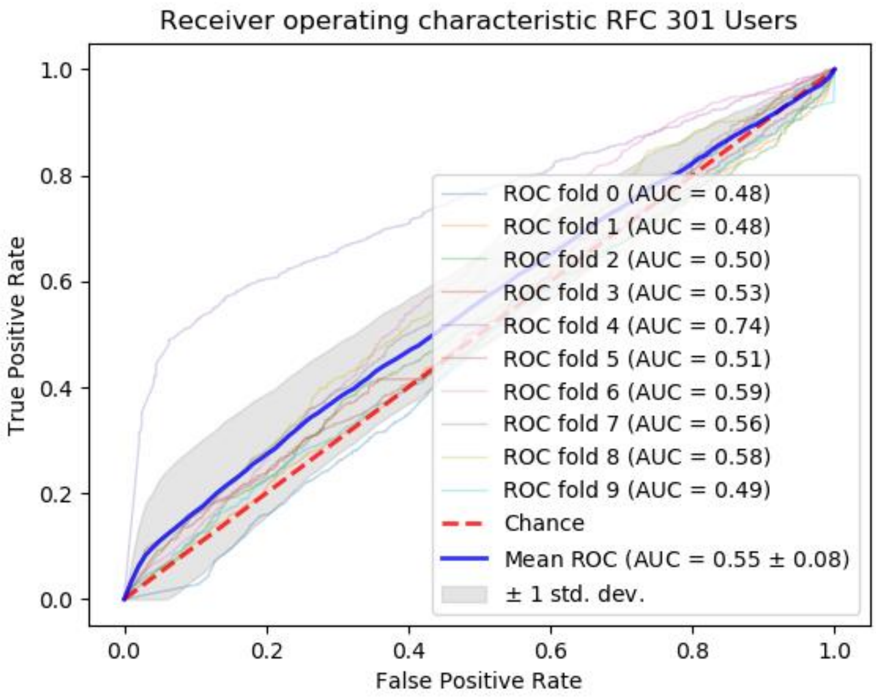

Supplementary Figure S10 10-folds ROC curve, AUC RFC, Rather absent Tinnitus Users

Support Vector Machine (SVM)

All Users:

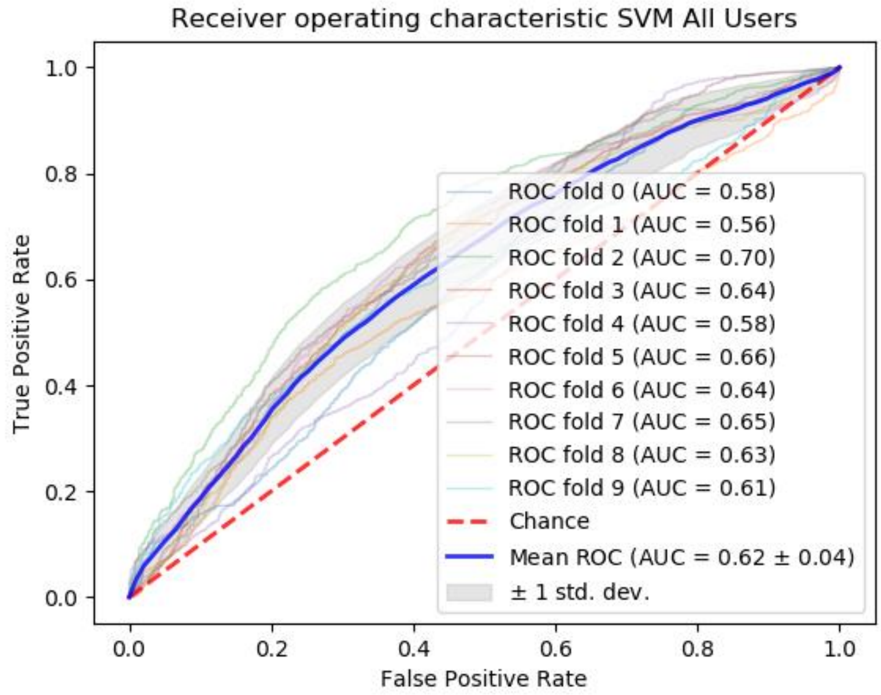

Supplementary Figure S11 10-folds ROC curve, AUC SVM, All Users

Power Users:

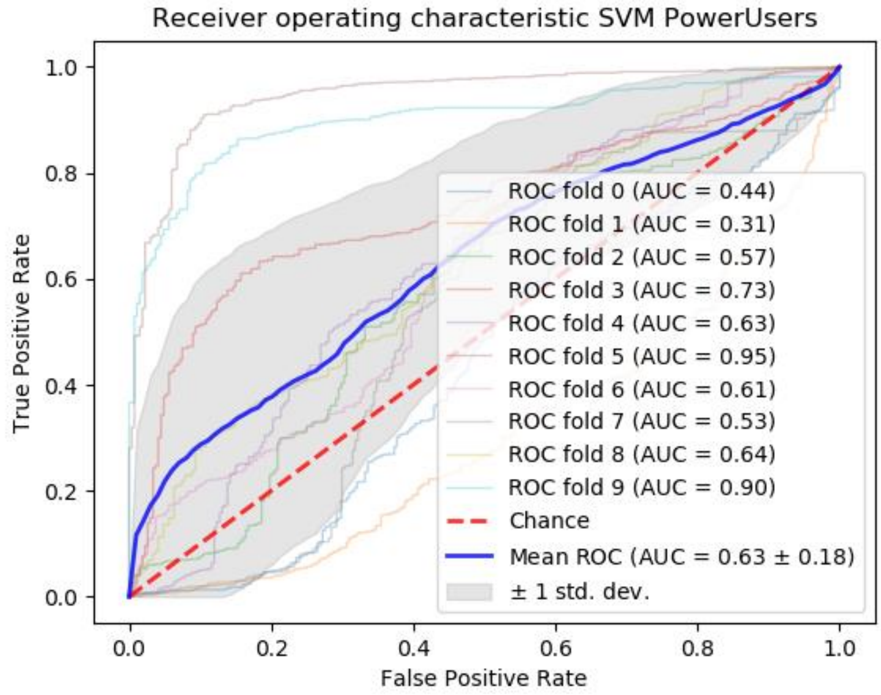

Supplementary Figure S12 10-folds ROC curve, AUC SVM, Power Users

Normal Users:

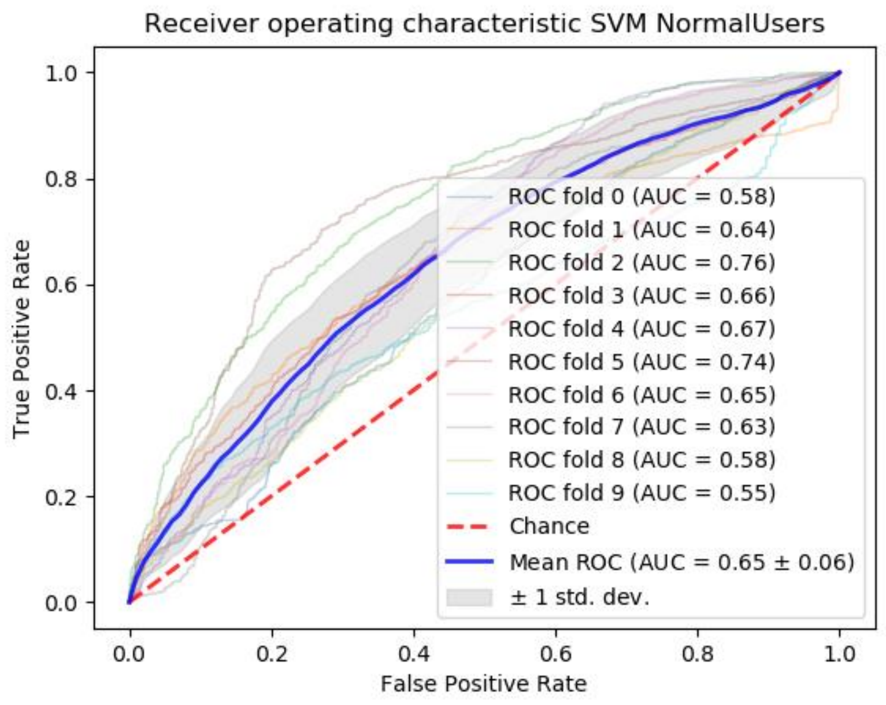

Supplementary Figure S13 10-folds ROC curve, AUC SVM, Normal Users

Non-permanent Tinnitus Users:

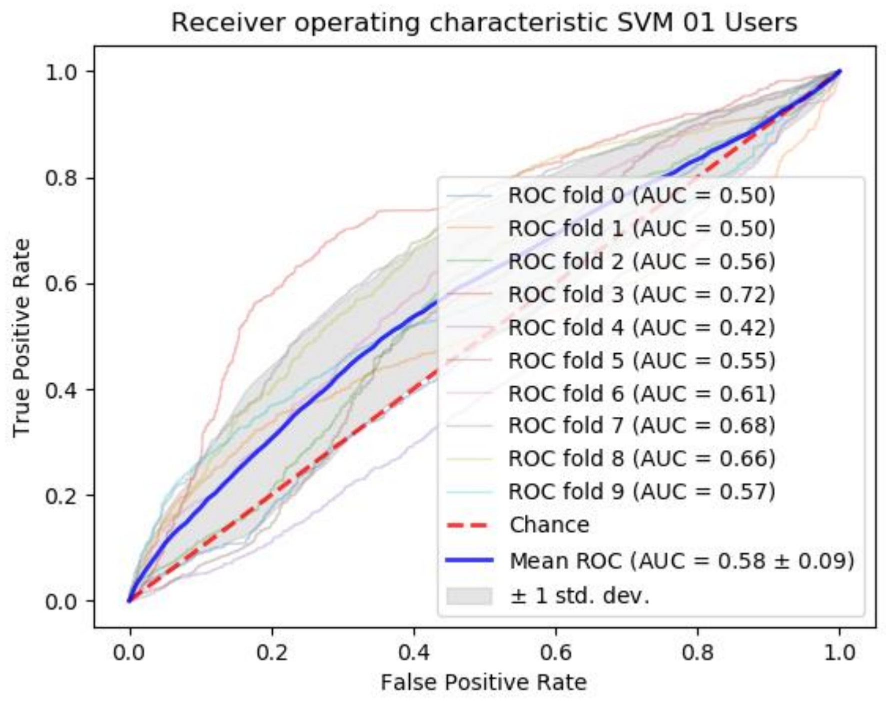

Supplementary Figure S14 10-folds ROC curve, AUC SVM, Non-permanent Tinnitus Users

Rather absent Tinnitus Users:

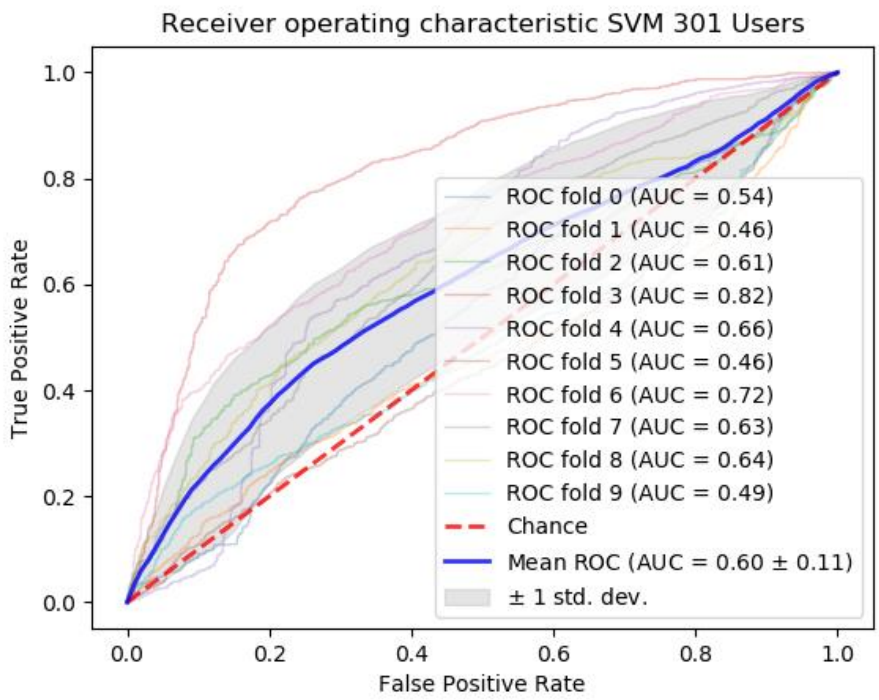

Supplementary Figure S15 10-folds ROC curve, AUC SVM, Rather absent Tinnitus Users

Complement Naive Bayes (CNB)

All Users:

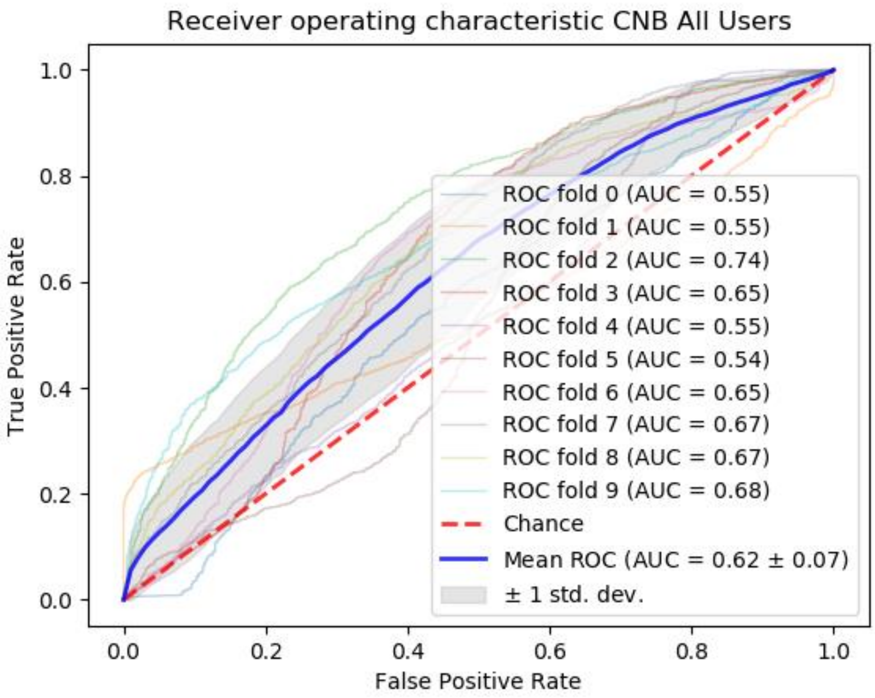

Supplementary Figure S16 10-folds ROC curve, AUC CNB, All Users

Power Users:

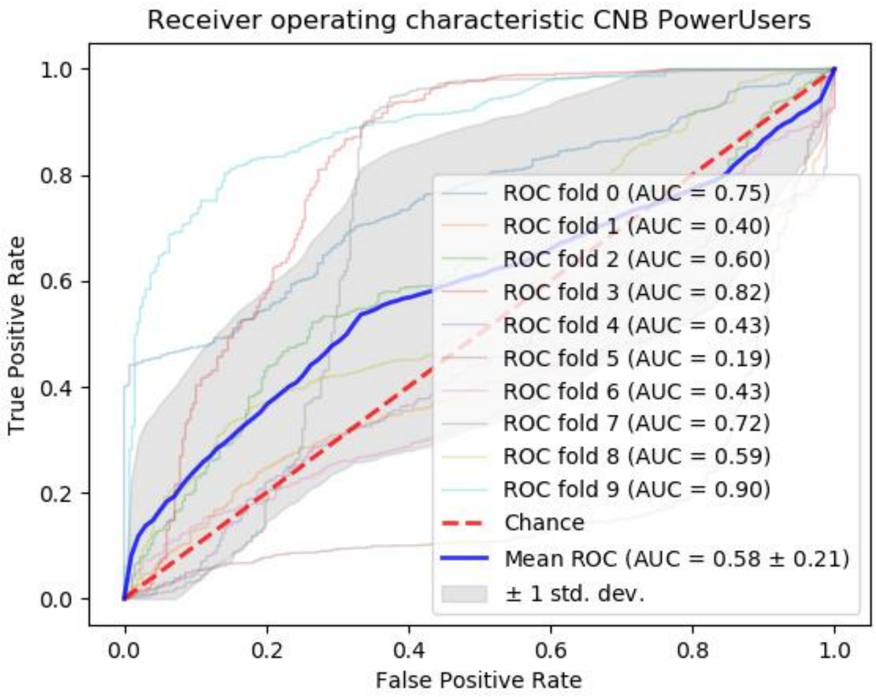

Supplementary Figure S17 10-folds ROC curve, AUC CNB, Power Users

Normal Users:

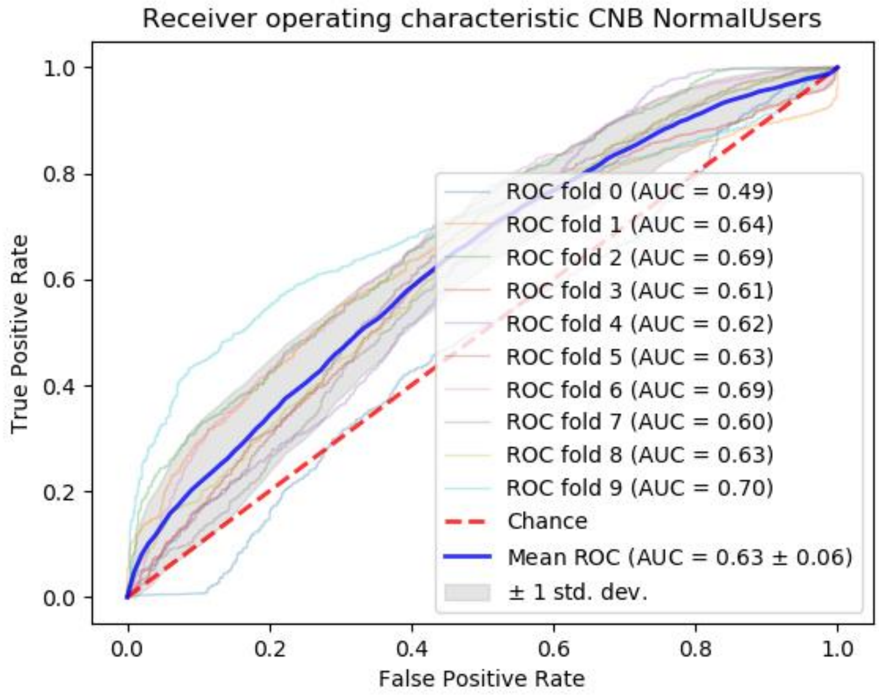

Supplementary Figure S18 10-folds ROC curve, AUC CNB, Normal Users

Non-permanent Tinnitus Users:

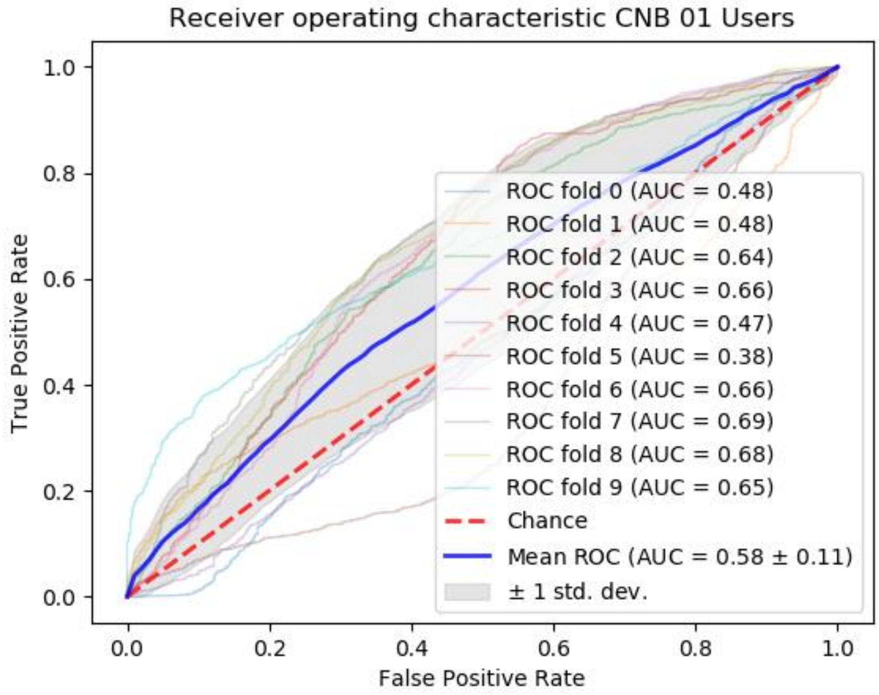

Supplementary Figure S19 10-folds ROC curve, AUC CNB, Non-permanent Tinnitus Users

Rather absent Tinnitus Users:

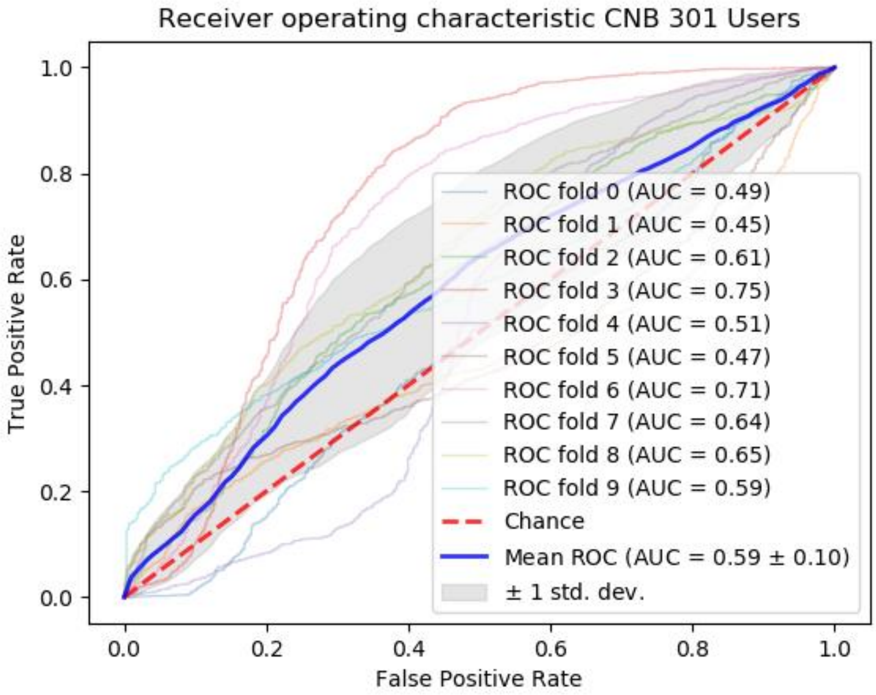

Supplementary Figure S20 10-folds ROC curve, AUC CNB, Rather absent Tinnitus Users

k-nearest neighbors (KNC)

All Users

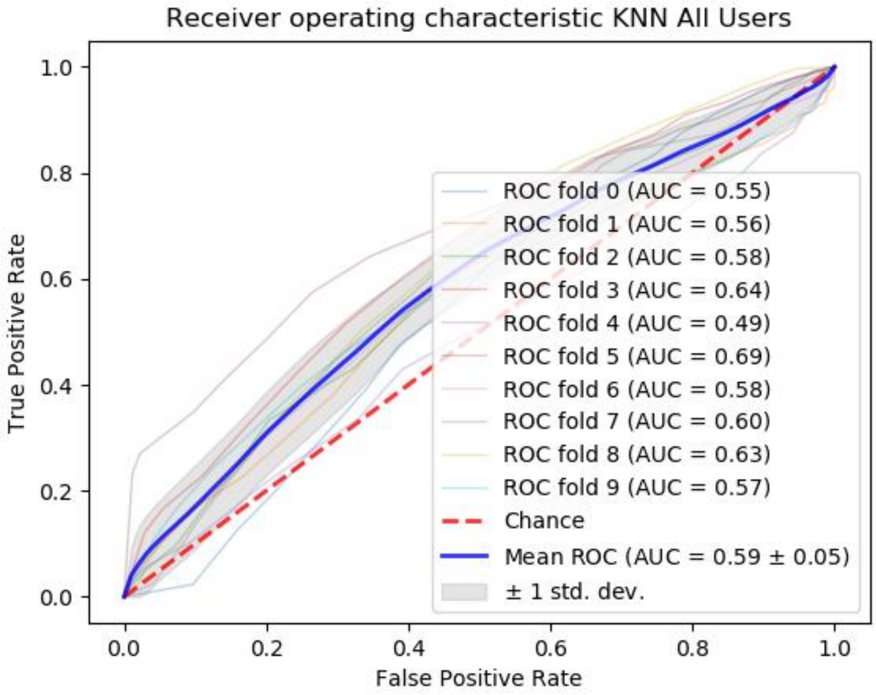

Supplementary Figure S21 10-folds ROC curve, AUC KNC, All Users

Power Users:

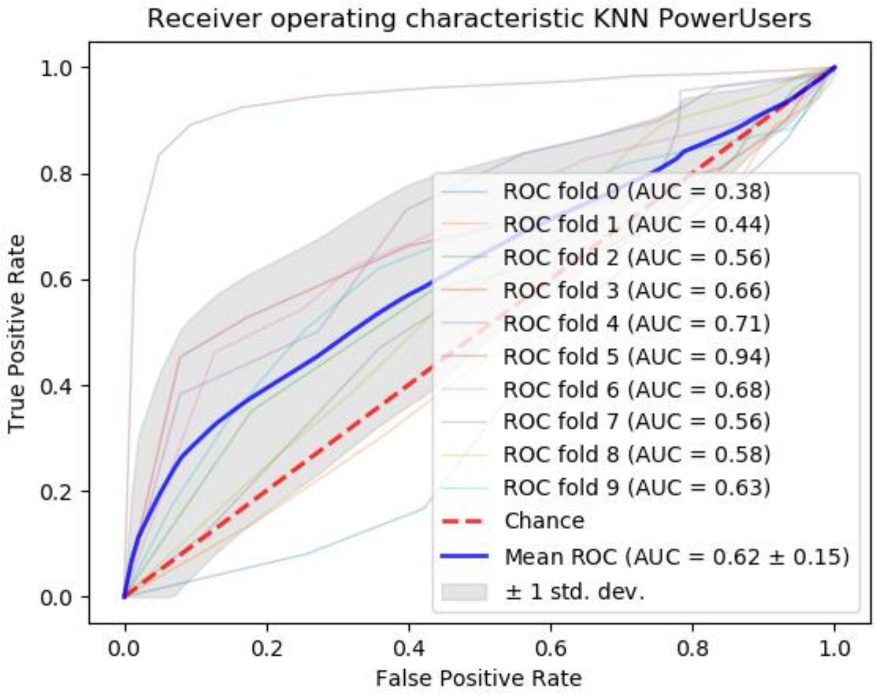

Supplementary Figure S22 10-folds ROC curve, AUC KNC, Power Users

Normal Users:

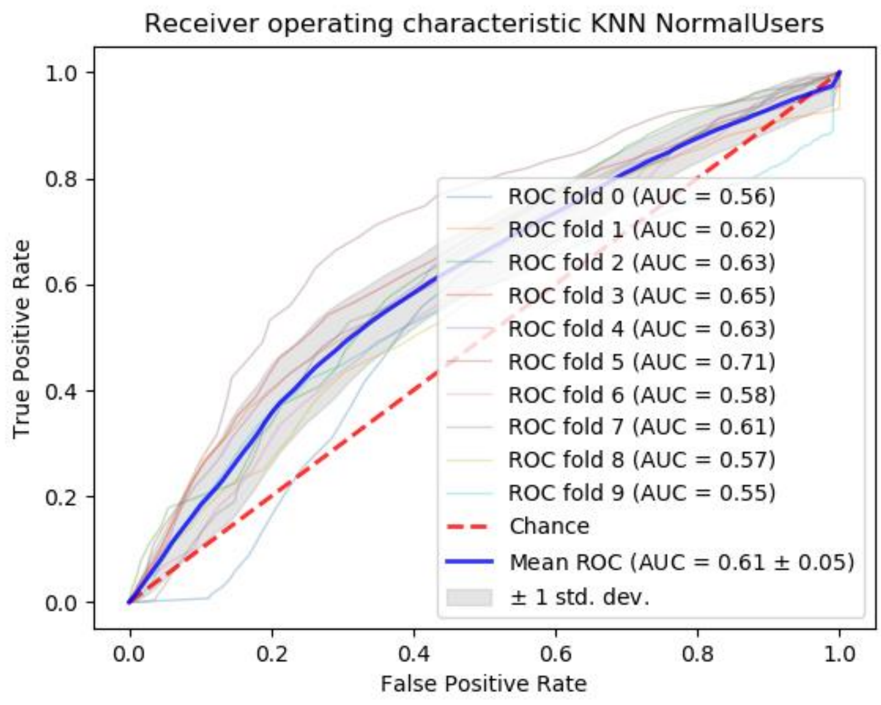

Supplementary Figure S23 10-folds ROC curve, AUC KNC, Normal Users

Non-permanent Tinnitus Users:

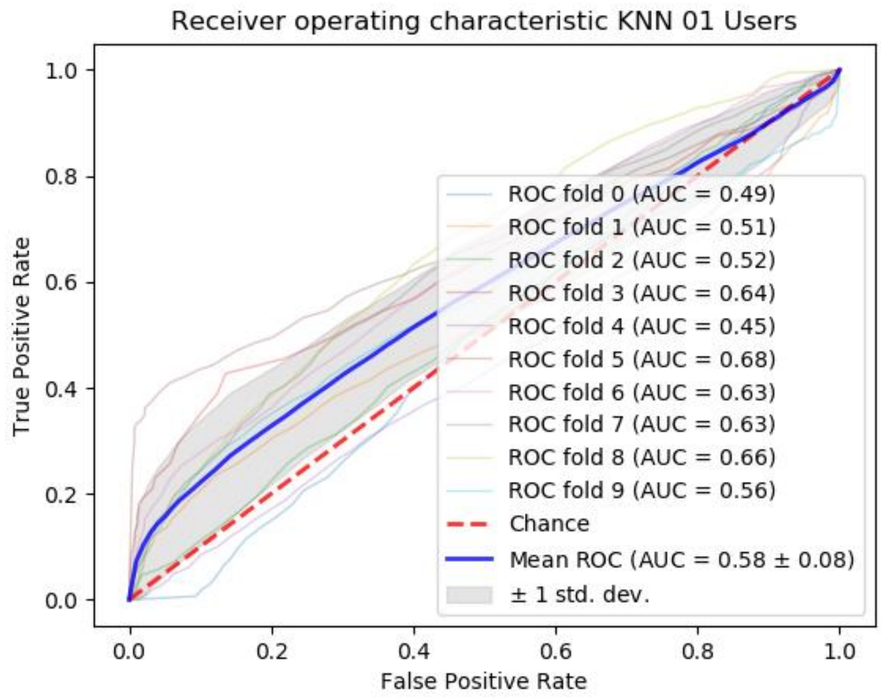

Supplementary Figure S24 10-folds ROC curve, AUC KNC, Non-permanent Tinnitus Users

Rather absent Tinnitus Users:

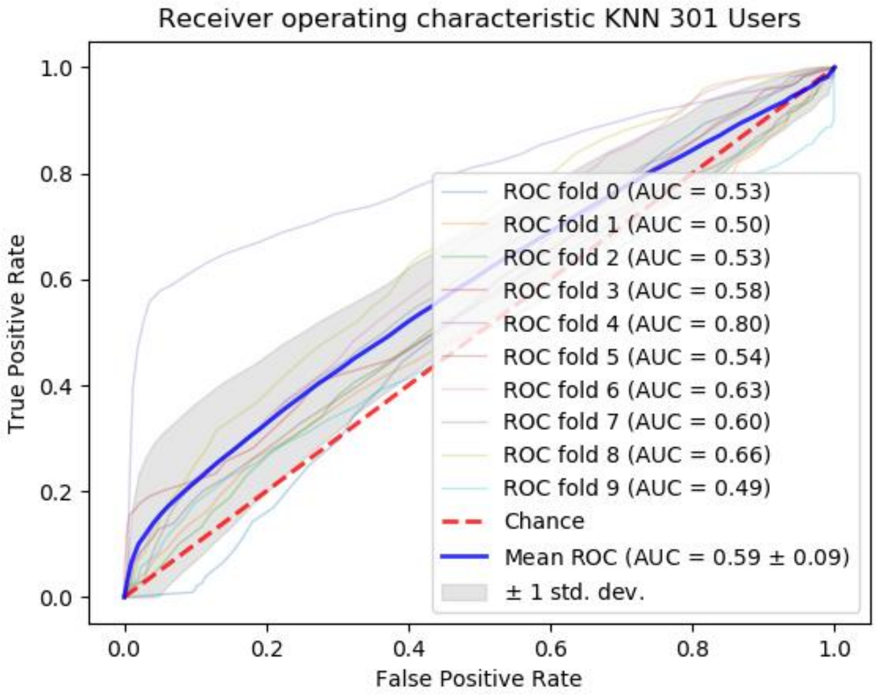

Supplementary Figure S25 10-folds ROC curve, AUC KNC, Rather absent Tinnitus Users

Logistic Regression (LRC)

All Users:

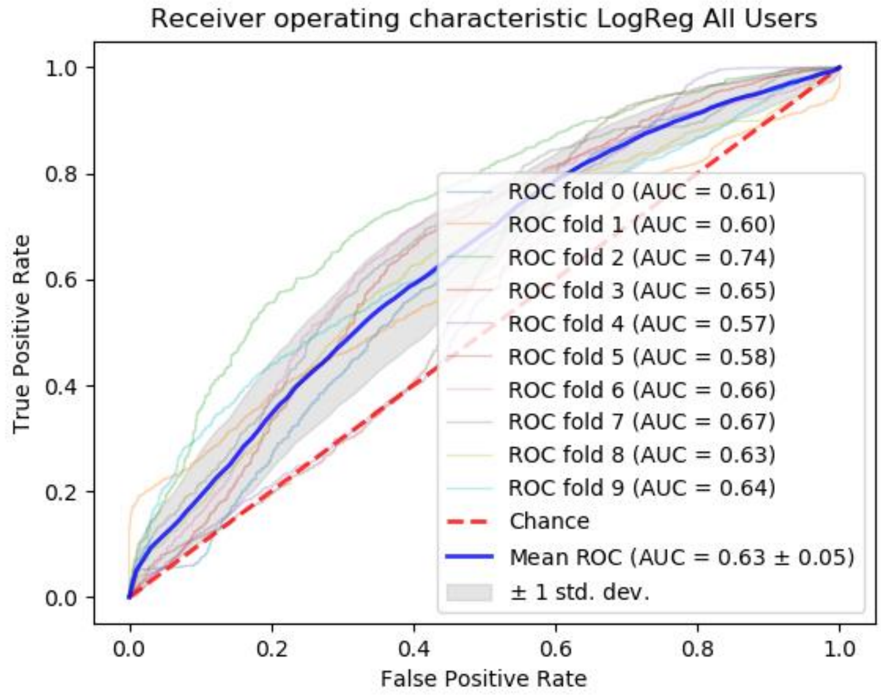

Supplementary Figure S26 10-folds ROC curve, AUC LRC, All Users

Power Users:

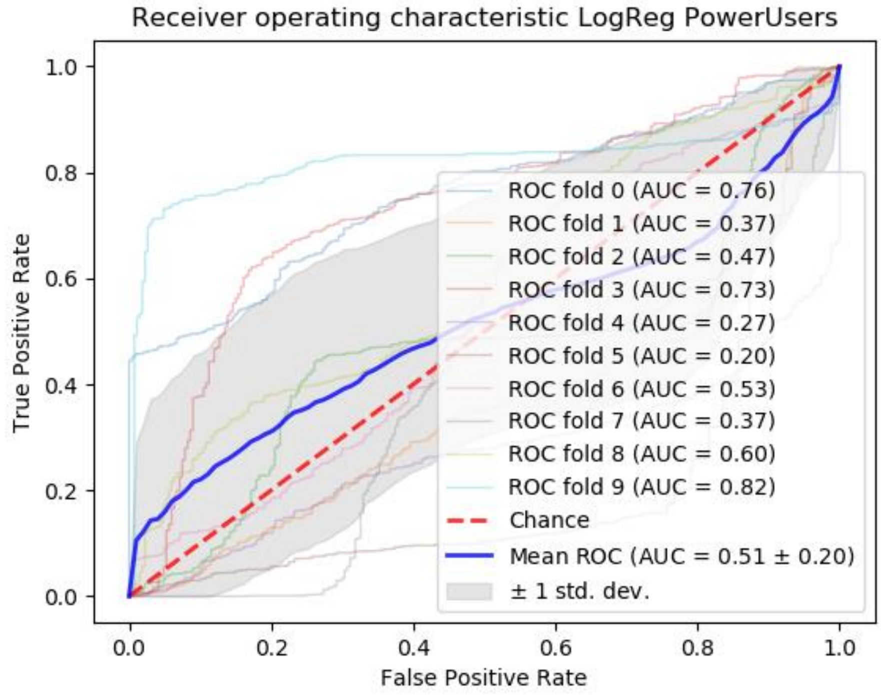

Supplementary Figure S27 10-folds ROC curve, AUC LRC, Power Users

Normal Users:

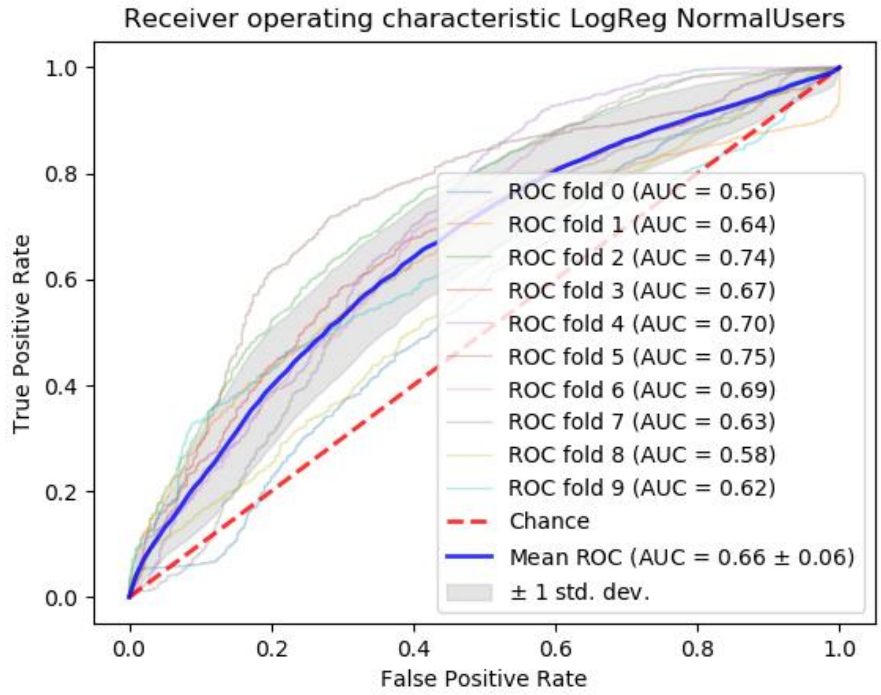

Supplementary Figure S28 10-folds ROC curve, AUC LRC, Normal Users

Non-permanent Tinnitus Users:

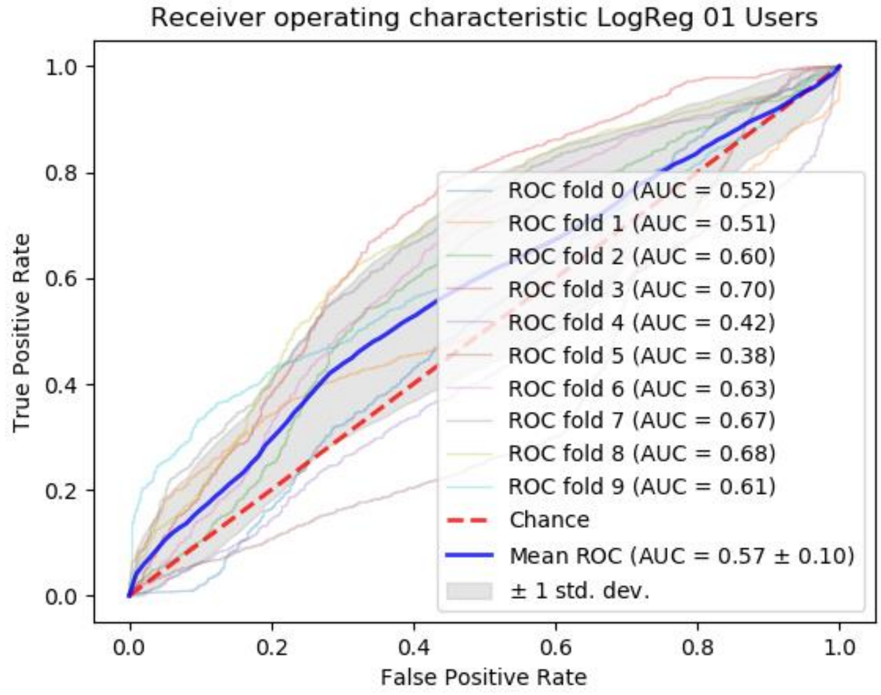

Supplementary Figure S29 10-folds ROC curve, AUC LRC, Non-permanent Tinnitus Users

Rather absent Tinnitus Users:

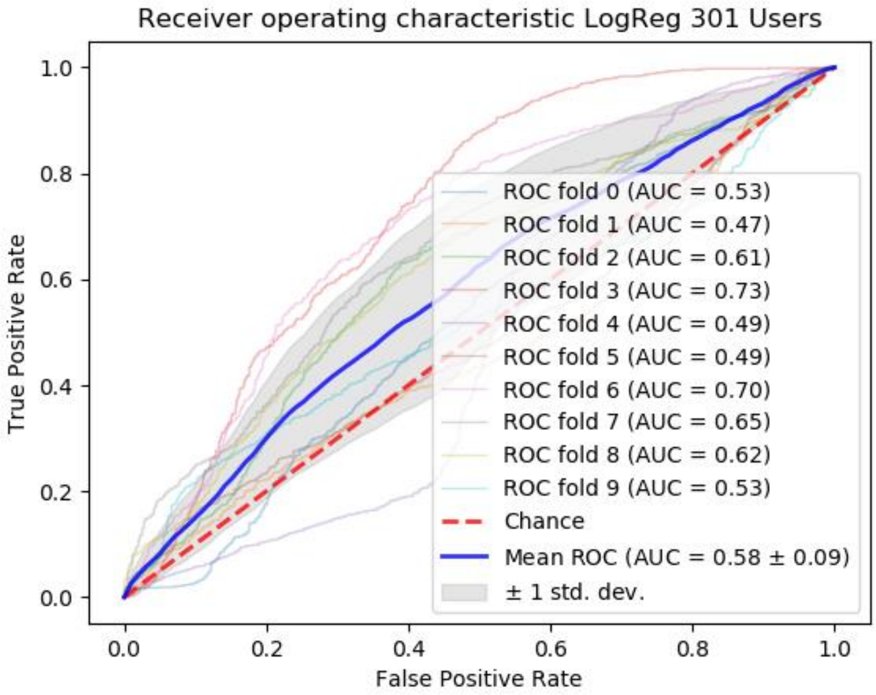

Supplementary Figure S30 10-folds ROC curve, AUC LRC, Rather absent Tinnitus Users

Multi-layer perceptron (MLP)

All Users:

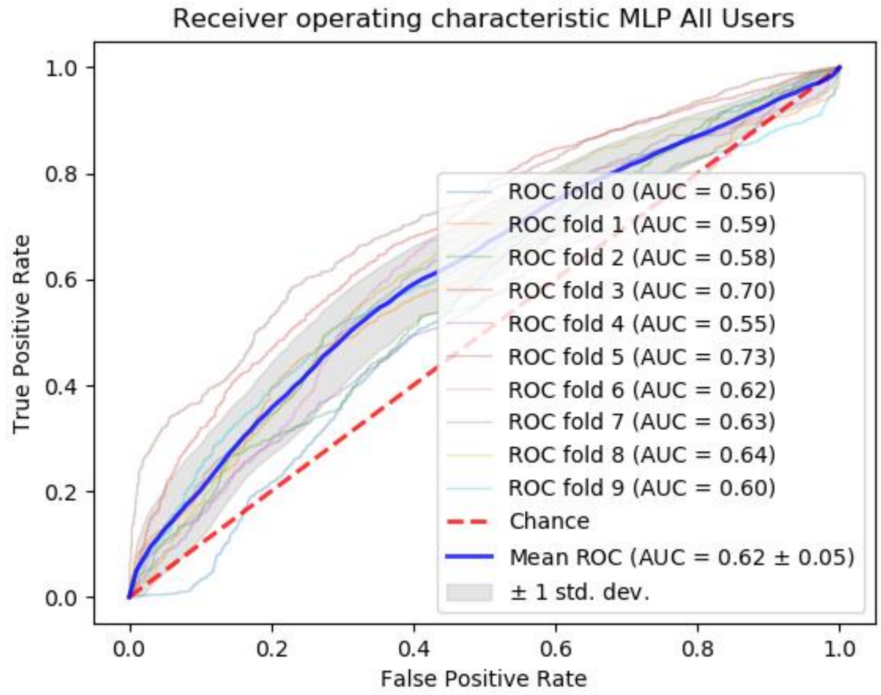

Supplementary Figure S31 10-folds ROC curve, AUC MLP, All Users

Power Users:

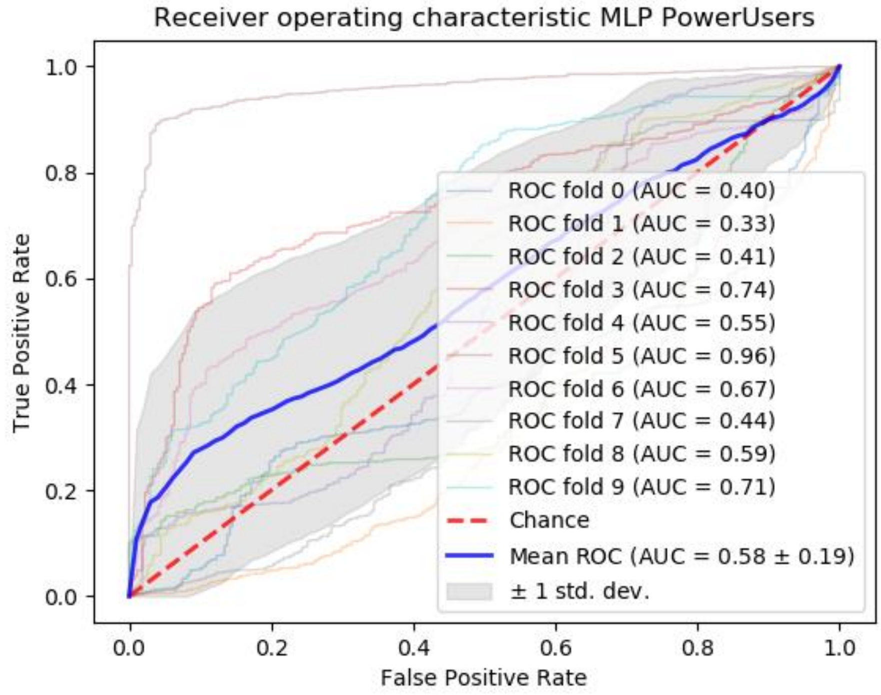

Supplementary Figure S32 10-folds ROC curve, AUC MLP, Power Users

Normal Users:

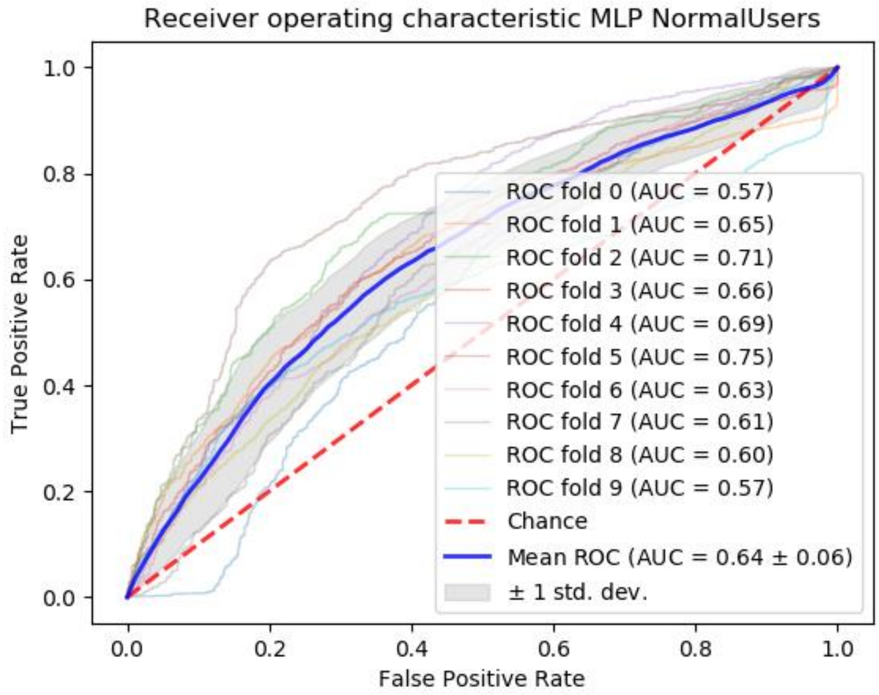

Supplementary Figure S33 10-folds ROC curve, AUC MLP, Normal Users

Non-permanent Tinnitus Users:

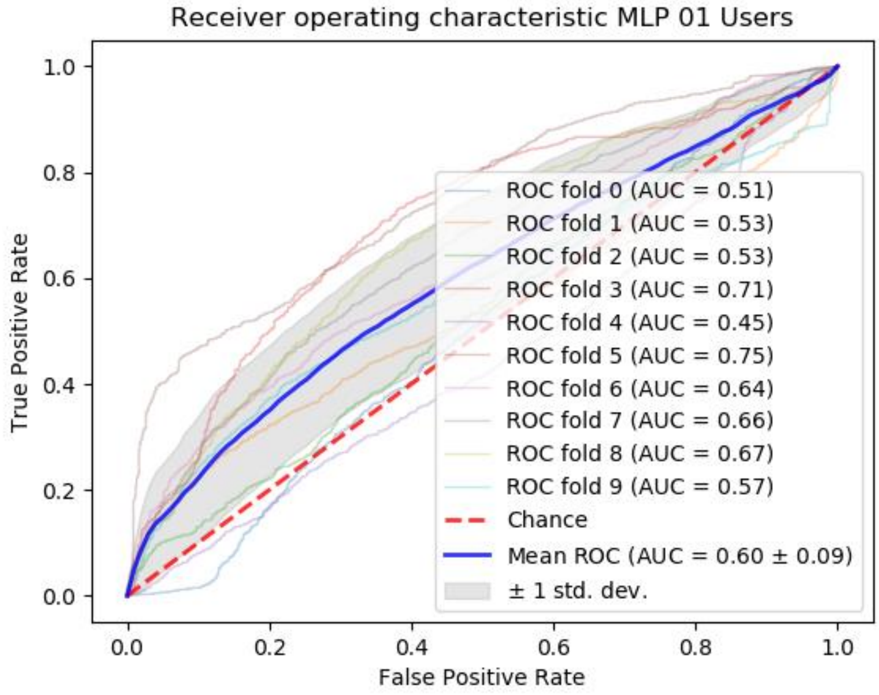

Supplementary Figure S34 10-folds ROC curve, AUC MLP, Non-permanent Tinnitus Users

Rather absent Tinnitus Users:

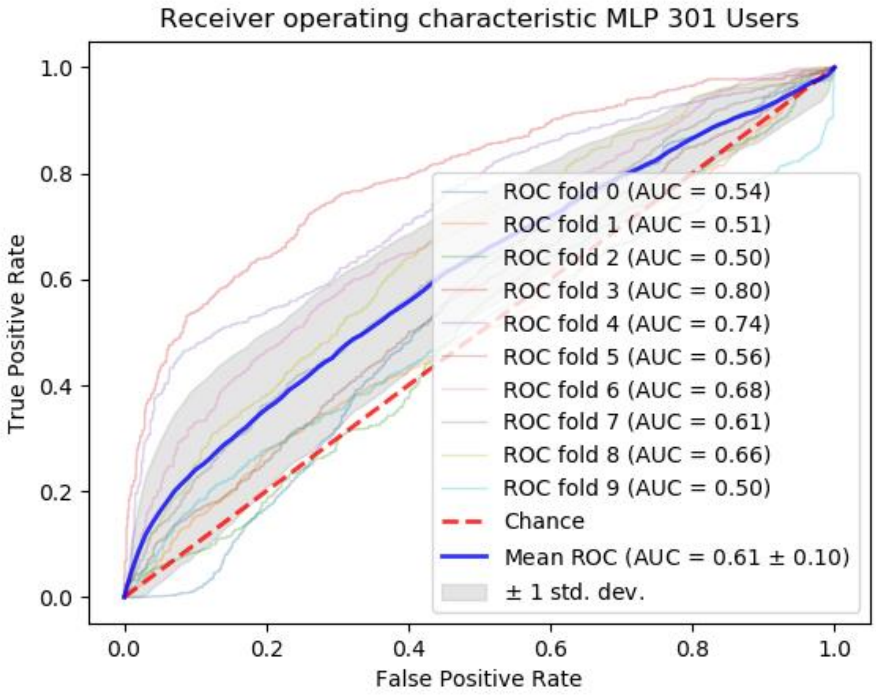

Supplementary Figure S35 10-folds ROC curve, AUC MLP, Rather absent Tinnitus Users

Extreme Gradient Boosting (XGB)

All Users:

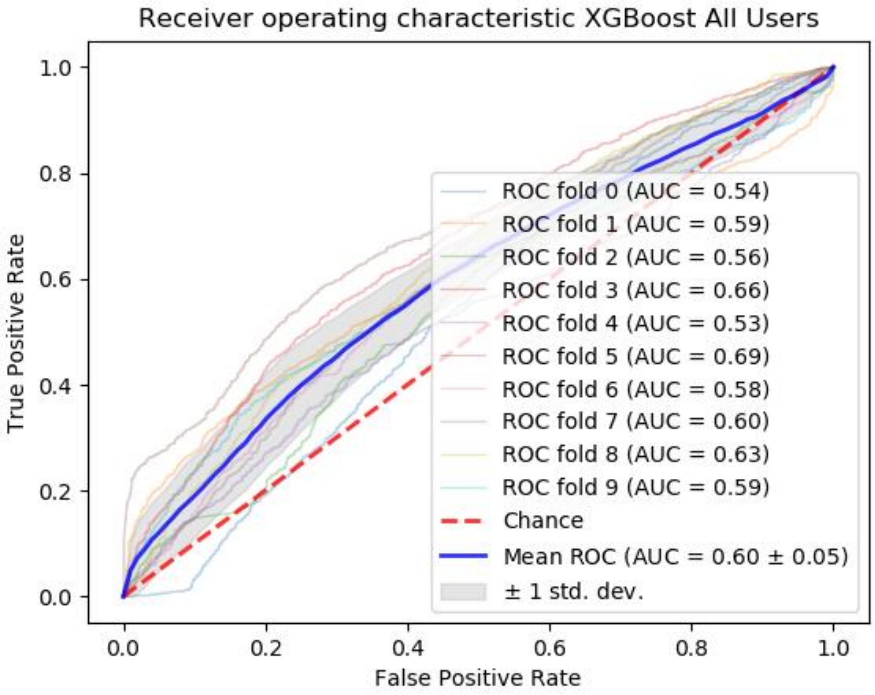

Supplementary Figure S36 10-folds ROC curve, AUC XGB, All Users

Power Users:

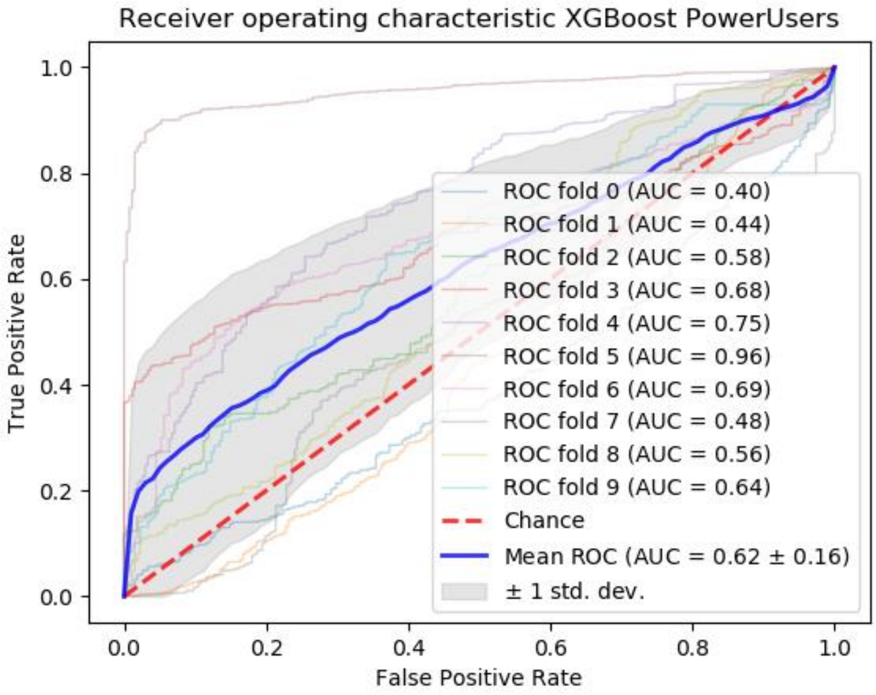

Supplementary Figure S37 10-folds ROC curve, AUC XGB, Power Users

Normal Users:

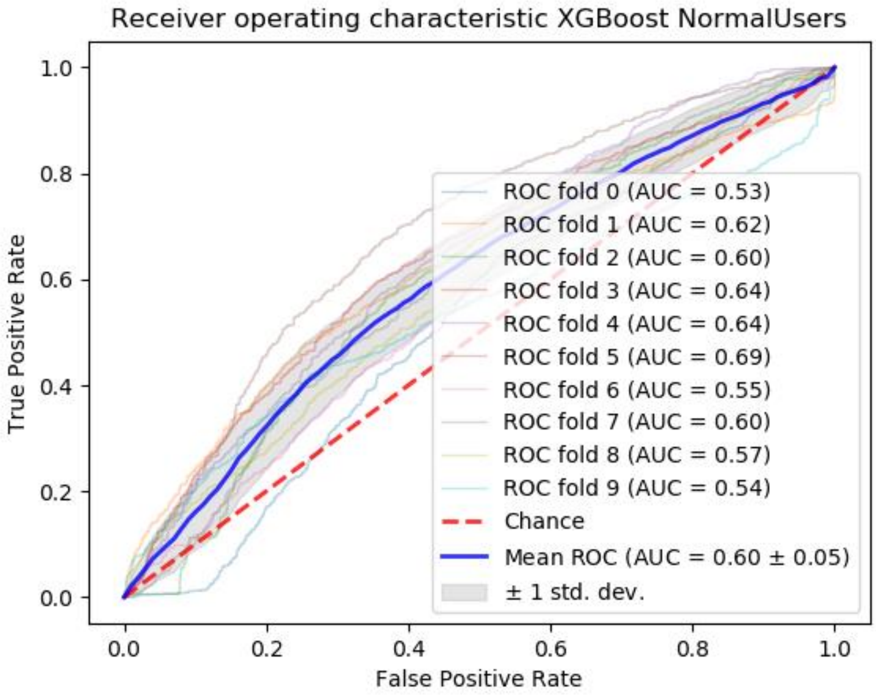

Supplementary Figure S38 10-folds ROC curve, AUC XGB, Normal Users

Non-permanent Tinnitus Users:

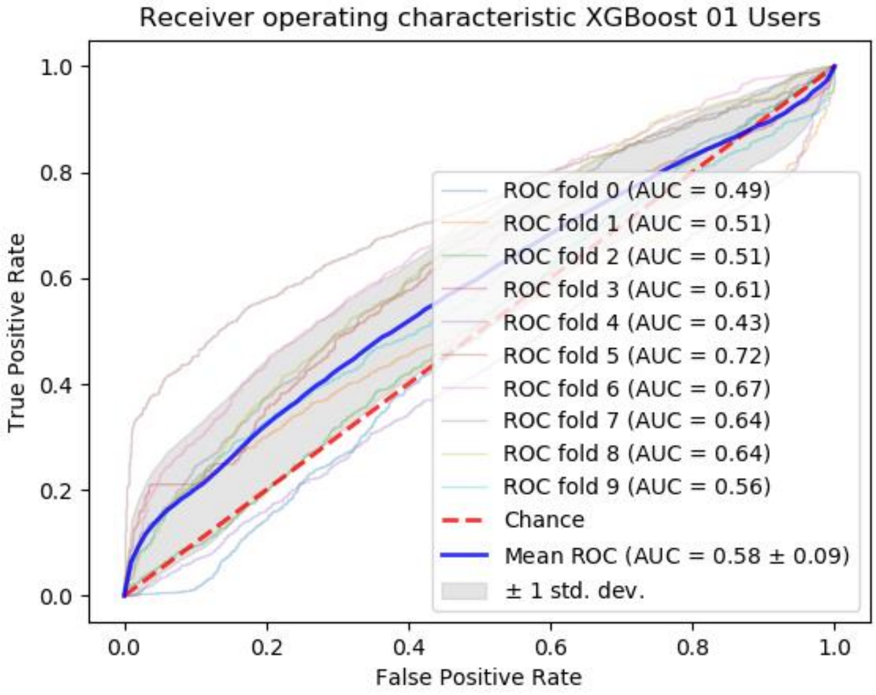

Supplementary Figure S39 10-folds ROC curve, AUC XGB, Non-permanent Tinnitus Users

Rather absent Tinnitus Users:

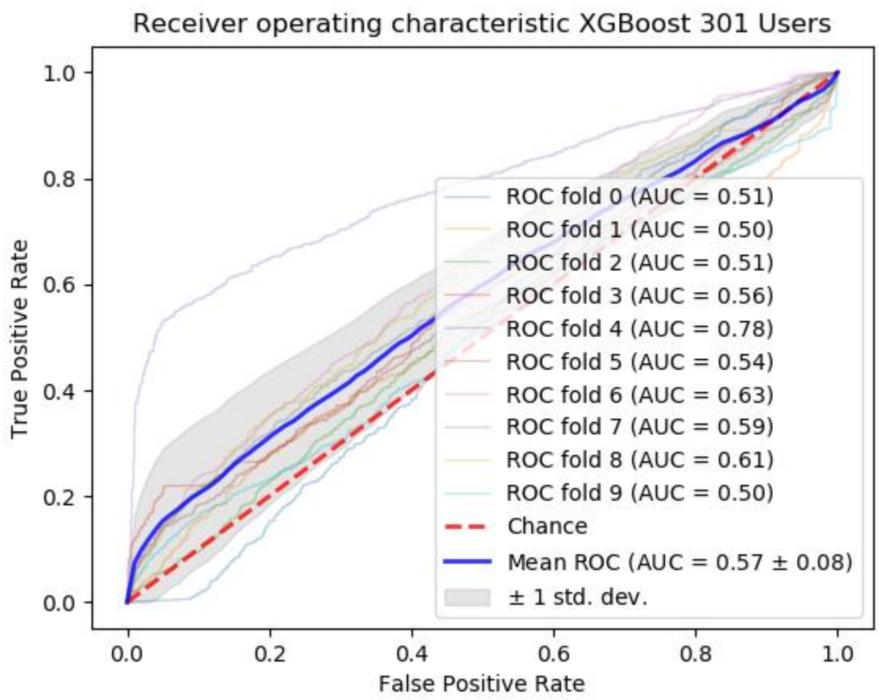

Supplementary Figure S40 10-folds ROC curve, AUC XGB, Rather absent Tinnitus Users
